# Differential Mucosal Microbiome Profiles across Stages of Human Colorectal Cancer

**DOI:** 10.3390/life11080831

**Published:** 2021-08-13

**Authors:** Mingqing Zhang, Yongming Lv, Shaobin Hou, Yanfei Liu, Yijia Wang, Xuehua Wan

**Affiliations:** 1 Nankai University School of Medicine, Nankai University, Tianjin 300071, China; mqzhang@nankai.edu.cn; 2 Tianjin Union Medical Center, Nankai University, Tianjin 300121, China; yongminglv_9274@163.com (Y.L.); lyanfei134@163.com (Y.L.); 3 Advanced Studies in Genomics, Proteomics and Bioinformatics, University of Hawaii, Honolulu, HI 96822, USA; shaobin@hawaii.edu; 4 Graduate School, Tianjin University of Traditional Chinese Medicine, Tianjin 301617, China; 5 TEDA Institute of Biological Sciences and Biotechnology, Nankai University, TEDA, Tianjin 300071, China

**Keywords:** gut microbiota, colorectal cancer, 16S rRNA sequencing

## Abstract

Emerging evidences link gut microbiota to colorectal cancer (CRC) initiation and development. However, the CRC stage- and spatial-specific bacterial taxa were less investigated, especially in a Chinese cohort, leading to our incomplete understanding of the functional roles of gut microbiota in promoting CRC progression and recurrence. Here, we report the composition and structure of gut microbiota across CRC stages I, II and III, by analyzing the gut mucosal microbiomes of 75 triplet-paired samples collected from on-tumor, adjacent-tumor and off-tumor sites and 26 healthy controls. We observed tumor-specific pattern of mucosal microbiome profiles as CRC progressed and identified ten bacterial taxa with high abundances (>1%) as potential biomarkers for tumor initiation and development. *Peptostreptococcus* and *Parvimonas* can serve as biomarkers for CRC stage I. *Fusobacterium*, *Streptococcus*, *Parvimonas*, *Burkholderiales*, *Caulobacteraceae*, *Delftia* and *Oxalobacteraceae* can serve as biomarkers for CRC stage II, while *Fusobacterium*, *Burkholderiales*, *Caulobacteraceae*, *Oxalobacteraceae*, *Faecalibacterium* and *Sutterella* can serve as biomarkers for CRC stage III. These biomarkers classified CRC stages I, II and III distinguished from each other with an area under the receiver-operating curve (AUC) > 0.5. Moreover, co-occurrence and co-excluding network analysis of these genera showed strong correlations in CRC stage I, which were subsequently reduced in CRC stages II and III. Our findings provide a reference index for stage-specific CRC diagnosis and suggest stage-specific roles of *Peptostreptococcus*, *Fusobacterium*, *Streptococcus* and *Parvimonas* in driving CRC progression.

## 1. Introduction

Colorectal cancer (CRC) is the third leading cause of cancer-related deaths after lung and breast cancers, leading to a public health issue worldwide. Emerging evidences implicate that, besides genetic and epigenetic factors, the human gut microbiota is a primary driver of inflammation in the colon and is strongly linked to CRC risk [1,2]. Based on the 16S ribosomal RNA (rRNA) gene and metagenomic sequencing, the compositions of gut mucosal and stool microbiota in CRC patients show significant differences from those in healthy individuals, suggesting that microbiome profiles plays pathological roles in CRC progression [3]. Several species associated with microbiome profiles in CRC, such as *Fusobacterium nucleatum*, *Peptostreptococcus anaerobius*, *Bacteroides fragilis* and *Eubacterium rectale*, have been investigated for their physiological functions in driving colorectal carcinogenesis [4,5,6,7].

The intestinal niche contains various notable factors governing the composition of gut microbiota [8]. Compared to normal epithelial cells, cancer cells enhance expression levels of sialylation and high-mannose glycans, as well as decrease levels of fucosylation and highly branched N-glycans. The N-glycosylation of cancer cells spreads into the surrounding stroma at the invasive front of tumor, which is associated with CRC developmental stages [9]. In addition, tumor cells reprogram their metabolism and secrete metabolites to shape tumor microenvironment (TME). Immune and non-immune cells, such as macrophages, neutrophils and fibroblasts, migrate to TME and contribute to systemic inflammation and increased oxidative stress and fibrosis [10]. The compositions of immune and non-immune cells and cell-surface glycans that serve as the major carbon source available to intestinal microbiota may play roles in determining the composition and structure of intestinal microbiota. For example, *Faecalibacterium* spp. and *Eubacterium* spp., which belong to butyrate-producing bacteria, are well suited to colonize on normal intestinal mucosal surfaces [11]. In contrast, certain bacteria, such as *Fusobacterium nucleatum* [7] and *Clostridium leptum* [12], exhibit low ability to bind the intact wall of a healthy colon but are suitable to colonize in TME with bleeding and ruptured colon wall of adenoma or carcinoma. *Streptococcus gallolyticus* subsp. *gallolyticus* (*Sgg*) shows low adhesion ability to healthy colonocytes and remains relatively low abundance (2.5–15%) in the intestinal microbiota of human [13]. When a tumor forms, *Sgg* exploits its pilus and collagen-binding proteins to bind to collagen that is highly expressed on the surface of tumor tissues [14]. On the other hand, *Sgg* can utilize host particular metabolites such as glucose-3-phosphate and fructose-6-phosphate, which are derived from increased glycolysis in tumor cells, for its own proliferation [15]. Therefore, certain species in gut microbiota gain competitive advantage in persistence and proliferation in TME. As TME changes during CRC development, the structure alteration of gut mucosal microbiome and the consequent effect of gut microbiome profiles in CRC initiation and development remain unclear.

To investigate whether the composition of gut mucosal microbiome exhibits a stage-specific pattern in TME, we collected gut mucosal microbiomes from 7 patients diagnosed at CRC stage I, 37 at CRC stage II and 31 at CRC stage III in Tianjin Union Medical Center, China. For each patient, triplet-paired CRC samples were collected from on-tumor site, adjacent-tumor site and off-tumor site. Additional samples were collected from 26 healthy controls. Our data revealed severed profiles of microbiome structures and stage-specific bacterial taxa as potential biomarkers during CRC evolution.

## 2. Materials and Methods

### 2.1. Sample Collection and DNA Library Preparation

The gut microbiome samples were collected from 75 CRC patients diagnosed for CRC stages and 26 healthy people in Tianjin Union Medical Center. In total, 7, 37 and 31 patients were diagnosed with CRC in stages I, II and III, respectively. The information was listed in Table S1. As CRC patients diagnosed at stage I usually show no symptoms, not many patients come to the hospital for diagnosis. We did not take samples from CRC patients diagnosed at stage IV, because surgery cannot remove metastatic cancer cells (surgery is meaningless). Written informed consent was obtained from all participants prior to their inclusion in the study. All the protocols and procedures were approved by the Medical Ethics Board of Tianjin Union Medical Center. Typical meals in Tianjin include dumplings and noodles made from wheat or rice, meats and vegetables. Intestinal mucosal microbiome samples were collected from on-tumor site (T), adjacent-tumor site (P) and off-tumor site (N) in the same removed tissue of a given CRC patient. The average distance between T- and N-sites is above 20 cm, and the shortest distance is above 17 cm. The average distance between T- and P-sites is 2 cm. The healthy people were determined by colonoscopy diagnosis and their intestinal microbiome samples were collected as healthy controls (H).

Bacterial DNA was extracted and purified using ZR Fungal/Bacterial DNA kit (Zymo Research, Irvine, CA, USA) according to the manufacturer’s instructions, and quantified using Quant-iT PicoGreen dsDNA assay kit (Thermo Fisher, Sunnyvale, CA, USA). The 16S ribosomal RNA (rRNA) gene amplicon sequencing libraries targeting the V3-V4 region were prepared according to the Illumina manufactory manual. The amplification primers include forward primer, 5′TCGTCGGCAGCGTCAGATGTGTATAAGAGACAGCCTACGGGNGGCWGCAG and reverse primer 5′GTCTCGTGGGCTCGGAGATGTGTATAAGAGACA GGACTACHVGGGTATCTAATCC, according to the Illumina manufactory manual. The amplified DNA libraries were purified using AMPure XP beads (Beckman Coulter, Fullerton, CA, USA) and quantified using Quant-iT PicoGreen dsDNA assay kit (Thermo Fisher, Sunnyvale, CA, USA).

### 2.2. OTU Picking and Analysis of 16S rRNA Amplicons

The amplicon libraries were bidirectionally sequenced (2 × 300 bp) on Illumina MiSeq platform. The summary of reads information was listed in Table S2. Quality control and filtering of raw sequencing reads were carried out using FastQC (https://www.bioinformatics.babra ham.ac.uk/projects/fastqc/, accessed on 5 June 2019). The filtered paired-end reads were assembled using PandaSeq v2.10 [16] with default parameters. De novo OTU (Operational Taxonomic Unit) picking, taxonomic assignment and diversity analyses were carried out using QIIME v1.9.1 with Greengenes database v13.8 (http://qiime.org/home_static/dataFiles.html, accessed on 5 June 2019) [17]. In brief, assembled sequences were clustered against one another without external reference sequence and de novo OTUs were picked using a similarity threshold of 97%, which is commonly used to define bacterial species. Chimera detection and filtering were performed using USEARCH 6.1. Next, taxonomy was assigned to OTU representative sequences. Alpha diversities were calculated using make_rarefaction_plots.py command from the QIIME pipeline. The 16S rRNA sequencing reads have been submitted to the NCBI (National Center for Biotechnology Information) SRA (Sequence Read Archive) database under accession number PRJNA606879.

### 2.3. Analyses of Differential Abundances of Gut Mucosal Microbiota during CRC Development

The composition and structure differences of gut mucosal microbiota in different CRC stages and tissue-specific sites were initially analyzed using principal component analysis (PCA). The PCA was performed on R v4.0.3 using factoextra package v1.0.7 [18]. Relative abundance of bacterial taxa was determined using arcsine square root [3]. The genera with relative abundances >0.1% identified in tumor-specific sites were analyzed for Venn diagram [19]. To reveal abundance patterns of intestinal microbiota as CRC progressed, the change trends of microbial abundances in tumor-specific sites were used to classify the genera to four groups (group I: stage I > II > III; group II: stage I < II < III; group III: stage I > II < III; group IV: stage I < II > III). Heatmap was visualized using R command heatmap. Specific genera were selected to compare their abundances in the three sites (N-, P- and T-sites) between CRC stages. To compare the abundances of oral bacteria from individuals with CRC to their abundances in intestinal, the genera of oral microbiota with top abundances from Flemer et al. were used to retrieve and compare the microbial abundances on tumor-specific sites at CRC stages. The core microbiome and the linear discriminant analysis (LDA) effect size (LEfSe) were analyzed using online MicrobiomeAnalyst (https://dev.microbiomeanalyst.ca/MicrobiomeAnalyst/home.xhtml, accessed on 16 May 2021) [20,21].

### 2.4. Co-Occurrence and Co-Excluding Correlation Network Analysis

To construct microbial correlation network, Spearman’s Rank correlation coefficient matrix of the abundances of identified genera were calculated using an R package Hmisc v4.5.0 (Harrell, Vanderbilt University School of Medicine, Nashville, TN, USA). Microbial correlation network analysis was carried out using Cytoscape 3.7.2 [22]. The correlation strengths of co-occurrence and co-excluding interactions were determined using correlation coefficient values (r > 0.2 or r < −0.2).

### 2.5. Statistical Analysis

Statistical analyses were performed using statistical analysis in social science program (SPSS). The statistical significances of multiple sample comparisons were calculated using one-way ANOVA with Kruskal–Wallis test. *, *p* < 0.05; **, *p* < 0.01; ***, *p* < 0.001; ****, *p* < 0.0001.

## 3. Results

### 3.1. Gut Mucosal Microbiome Profiles during CRC Evolvement

**Figure 1:**
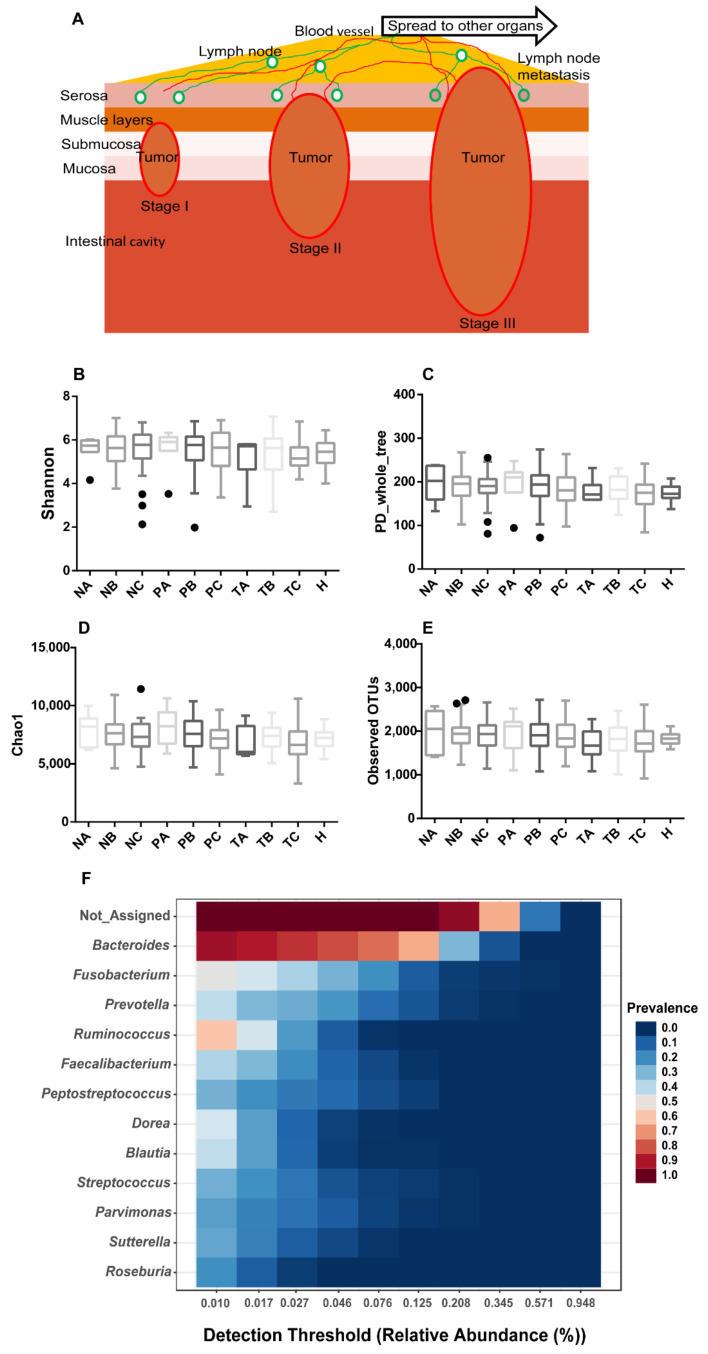
Microbial alpha-diversities showing no significant difference in on-tumor (T), adjacent-tumor (P) and off-tumor (N) sites during CRC development and healthy controls (H). (**A**) Schematic diagram showing CRC developmental process from stage I to stage III. Invasion depth increases during CRC development and lymph node metastasis occurs in CRC stage III leading to distant metastasis. (**B**). Alpha diversity evaluated using Shannon diversity index. (**C**). Alpha diversity evaluated using PD whole tree index. (**D**). Alpha diversity evaluated using Chao1 index. (**E**). Alpha diversity evaluated using Observed OTU index. (**F**). Core microbiome analysis showing a limited number of genera prevalent across all the samples. Sample prevalence threshold is set up above 20%, and relative abundance threshold is set up above 0.01%. The heatmap colors represent the sample prevalence values. A: CRC stage I; B: CRC stage II; C: CRC stage III. N: off-tumor site; P: adjacent-tumor site; T: on-tumor site. PD: Phylogenetic Diversity. Alpha-diversity differences were compared using one-way ANOVA with Kruskal–Wallis test, *p* > 0.05.

As CRC evolves, tumor aberrantly grows at stages I and II, with the capacity to eventually invade and spread to other parts of the human body at stage III (Figure 1A). To evaluate the structure variation of spatiotemporal microbiome during CRC development, we assessed microbial alpha-diversity of biopsy samples collected from on-tumor (T), adjacent-tumor (P) and off-tumor (N) sites at CRC stages I, II and III. The 16S ribosomal RNA gene hypervariable V3-V4 regions were sequenced and analyzed for four α-diversity indices including Shannon, Phylogenetic Diversity (PD) whole tree, Chao1 and Observed OTU. Analysis of species variation based on the four metrics consistently showed that species diversities in specific intestinal sites of CRC patients diagnosed with different CRC stages and healthy controls were not significantly different (one-way ANOVA with Kruskal–Wallis test, *p* < 0.05) (Figure 1B–E). Although increased α-diversity in TME has been reported [23], TME is not always linked to increased α-diversity [12,24,25,26]. In addition, sampling bias and low sequencing depth miss detection of rare species in samples and worsen the accuracy for estimation of α-diversity, leading to artificial differences in α-diversity [27]. Our data suggest that CRC initiation and development are associated with composition alteration of internal species residing in intestinal niche instead of obtaining external invading species at any CRC stages.

We next investigated the core taxa that remained unchanged in their compositions across all the samples from patients and healthy controls based on sample prevalence (>20%) and relative abundance (0.01%). Besides bacteria with unassigned taxa, 12 genera were identified as core taxa across all the samples, including *Bacteroides*, *Fusobacterium*, *Prevotella*, *Ruminococcus*, *Faecalibacterium*, *Peptostreptococcus*, *Dorea*, *Blautia*, *Streptococcus*, *Parvimonas*, *Sutterella* and *Roseburia* (Figure 1F). Among them, only *Bacteroides* and *Ruminococcus* were prevalent in more than half of the samples (Figure 1F). These data suggest that, although the above α-diversity indices did not show significant variation in species composition between the compared conditions (Figure 1B–E), the core taxa prevalent in the samples were limited to a small number even with a low prevalence threshold (20%). Thus, the abundances of the majority of bacterial species varied in the samples collected from CRC patients and healthy controls.

**Figure 2:**
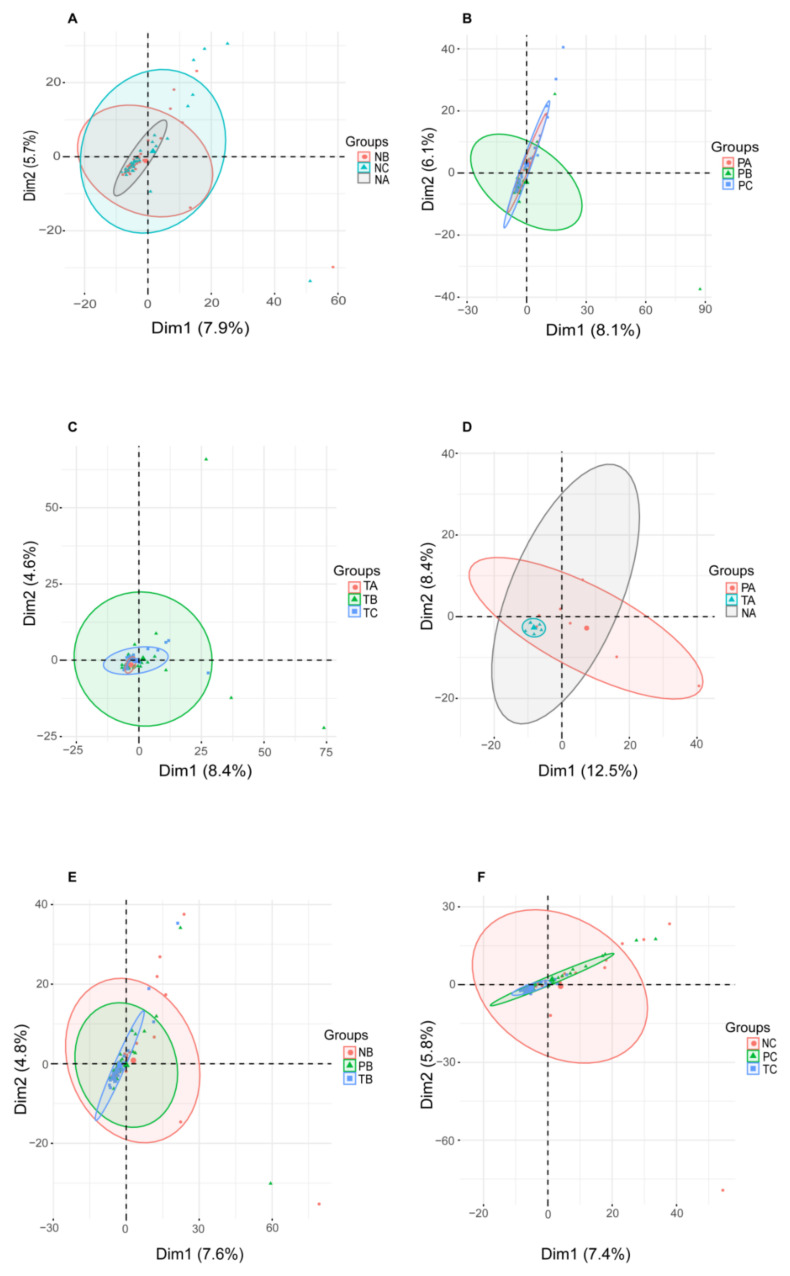
PCA showing microbiome profiles during CRC development. (**A**–**C**) PCA showing microbial diversity at off-tumor site (**A**), adjacent-tumor site (**B**) and on-tumor site (**C**) among CRC stages I, II and III. (**D**–**F**) showing microbial diversity at CRC stage I (**D**), II (**E**) and III (**F**) among off-tumor, adjacent-tumor and on-tumor sites. A: stage I; B: stage II; C: stage III. N: off-tumor site; P: adjacent-tumor site; T: on-tumor site. PCA: principle component analysis.

To assess the dissimilarity of microbiome structure diversities (beta-diversity) in specific intestinal sites during CRC development, we carried out principle component analysis (PCA) for genera abundances identified in T-, P- and N-sites of CRC patients diagnosed at stages I, II and III. In T-, P- and N-sites, the microbiome structure diversities showed differences at stages I, II and III (Figure 2A–C). Moreover, at stages I, II and III, the microbiome structure diversities in T-, P- and N-sites showed differences (Figure 2D–F). Thus, microbial abundances vary both spatially and temporally during CRC development, suggesting that intestinal microenvironment in CRC specific site and stage determines proliferation of the favored microbial species.

### 3.2. Genera with Top Abundances Showing Differential Abundances during CRC Progression

**Figure 3:**
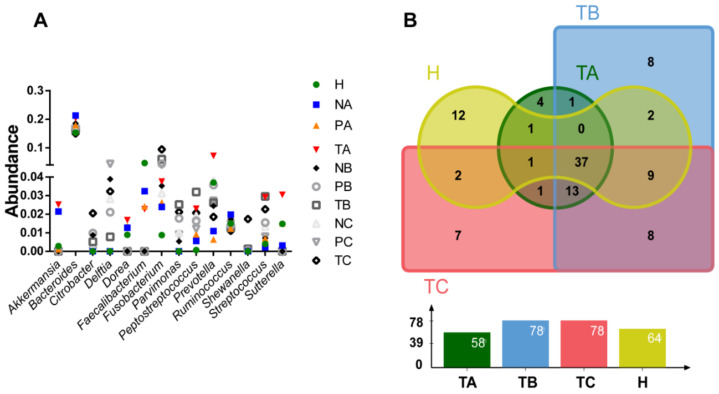
Comparison of the most abundant genera identified in each condition. (**A**). Abundance comparison of the top 10 genera in each condition. (**B**). Venn diagram visualizing comparison of genera with abundance >0.1% among CRC stages I, II and III at on-tumor site (T) and healthy controls. A: CRC stage I; B: CRC stage II; C: CRC stage III. N: off-tumor site; P: adjacent-tumor site; T: on-tumor site. H: healthy control.

To examine microbial differential abundances at genus level, we compared the top 10 most abundant genera from T-, P- and N-sites at CRC stages I, II and III and healthy controls (Figure 3A). A total of 14 genera were compared, including *Akkemansia*, *Bacteroides*, *Citrobacter*, *Delftia*, *Dorea*, *Faecalibacterium*, *Fusobacterium*, *Parvimonas*, *Peptostreptococcus*, *Prevotella*, *Ruminococcus*, *Shewanella*, *Streptococcus* and *Sutterella*. Among them, *Bacteroides* was the most abundant genus (~20%), showing little variation between conditions (Figure 3A). *Akkemanisia*, *Dorea*, *Prevotella*, *Streptococcus* and *Sutterella* showed the highest abundances in T-site at CRC stage I (Figure 3A). *Parvimonas* and *Peptostreptococcus* showed the highest abundances in T-site at CRC stage II (Figure 3A). *Citrobacter*, *Fusobacterium* and *Shewannella* showed the highest abundances in T-site at CRC stage III (Figure 3A). When the genera with abundances >0.1% in T-site at CRC stages I, II and III and those in healthy controls were compared, 37 were shared among all the four conditions (Figure 3B, Table S3), consistent with the above data that, although the abundances of species may vary as CRC develops, the alpha diversities showed no significant differences between conditions. Moreover, only 12, 4, 7 and 8 genera with abundances >0.1% were uniquely present in healthy controls and T-site at CRC stages I, II and III, respectively (Figure 3B, Table S3).

### 3.3. Altered Microbial Signatures in Different CRC Stages

**Figure 4:**
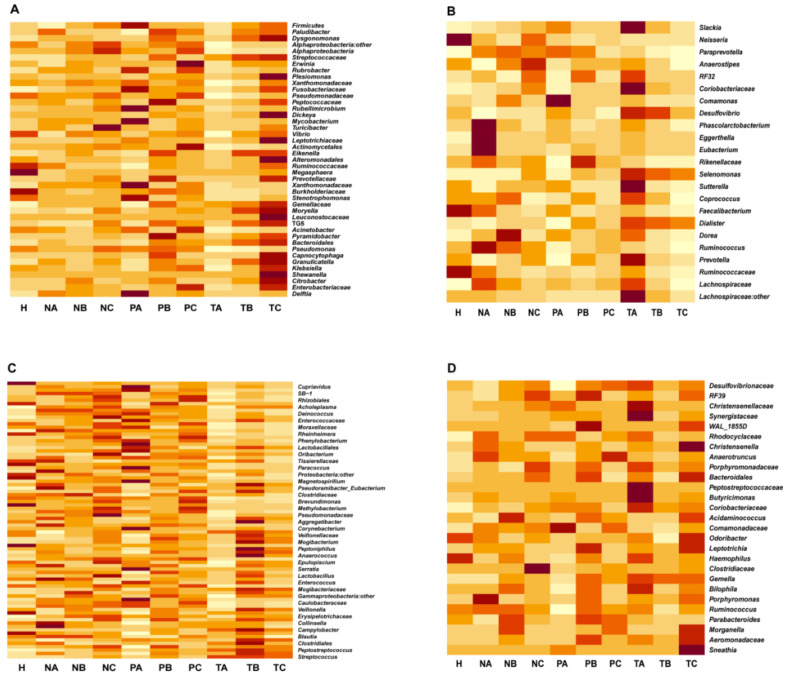
Microbiome abundances showing differential changing trends in on-tumor, adjacent-tumor and off-tumor sites when compared among CRC stages I, II and III. Heatmap visualizing abundance changes in all the conditions. (**A**). Genera abundances in on-tumor site at CRC stage I > II > III. (**B**). Genera abundances in on-tumor site at CRC stage I < II < III. (**C**). Genera abundances in on-tumor site at CRC stage I > II < III. (**D**). Genera abundances in on-tumor site at CRC stage I < II > III. A: CRC stage I; B: CRC stage II; C: CRC stage III. N: off-tumor site; P: adjacent-tumor site; T: on-tumor site. H: healthy control.

Next, we compared the microbiome changes in T-, P- and N-sites at CRC stages I, II and III, and healthy controls at genus level. The microbiome abundances did not show the same trends in the three sites when they were compared among the CRC stages (Figure 4). We classified the genera to four groups based on the change trends of their abundances in the T-site (group I: stage I > II > III, group II: stage I < II < III, group III: stage I > II < III, and group IV: stage I < II >III) (Figure 4A–D). In the T-site, groups I, II and III bacteria adapted well to proliferating in TME at CRC stage III, I, and II, respectively, whereas group IV bacteria showed the least adaptation to TME at CRC stage II (Figure 4A–D). For further understanding the physiological roles of bacteria identified in the four groups (Figure 4A–D), information regarding their metabolism and pathogenicity was listed in Table S4. These data suggest that CRC stage-specific microenvironment is associated with the enrichment of stage-specific species. As CRC develops, the abundances of some butyrate-producing bacteria (*Coprococcus* and *Anaerostipes*) decreased, and some (*Moryella* and *Megasphaera*) increased, whereas the abundances of pathogenic bacteria (*Citrobacter*, *Klebsiella* and *Stenotrophomonas*) increased. Notably, many of the latter were opportunistic pathogens that permeate broken intestinal wall in T-site to induce proinflammation in TME.

**Figure 5:**
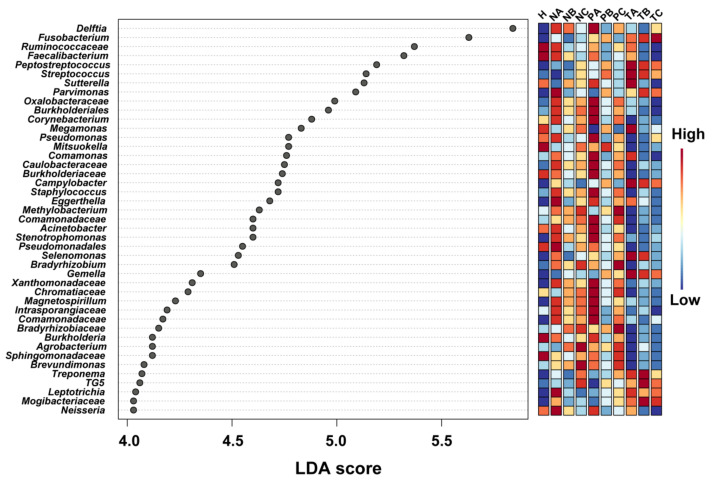
LEfSe analysis showing the genera or families with unknown genus with significant differential abundances in N-, P- or T-sites at CRC stages I, II and III. The colors in the heatmap represent the abundances of genera/families with unknown genus. A: CRC stage I; B: CRC stage II; C: CRC stage III. N: off-tumor site; P: adjacent-tumor site; T: on-tumor site. H: healthy control. LefSe: the linear discriminant analysis (LDA) effect size.

We next carried out the linear discriminant analysis (LDA) effect size (LEfSe) method to predict biomarkers that have potentials for diagnosis of CRC stages. By setting LDA score > 4.0, a total of 43 genera were identified with significant differential abundances in specific sites and CRC stages (*p* < 0.05) (Figure 5). Among them, *Fusobacterium*, *Peptostreptococcus*, *Streptococcus*, *Campylobacter*, *Gemella*, *Treponema*, TG5, *Leptotrichia* and *Mogibacteriaceae* showed higher abundances in T-site than in N- or P-sites, whereas the abundances of the other genera or families with unknown genus decreased in T-site compared to those in N- or P-sites (Figure 5). The abundances of *Fusobacterium* in T-site, as the only genus, consistently increased from CRC stage I to III (Figure 5). *Peptostreptococcus*, *Streptococcus*, *Campylobacter*, *Gemella* and *Leptotrichia* showed the highest abundances in T-site at CRC stage I with decreased abundances in T-site at CRC stages II and III (Figure 5). *Treponema*, TG5 and *Mogibacteriaceae* showed the highest abundances in T-site at CRC stage II (Figure 5).

**Figure 6:**
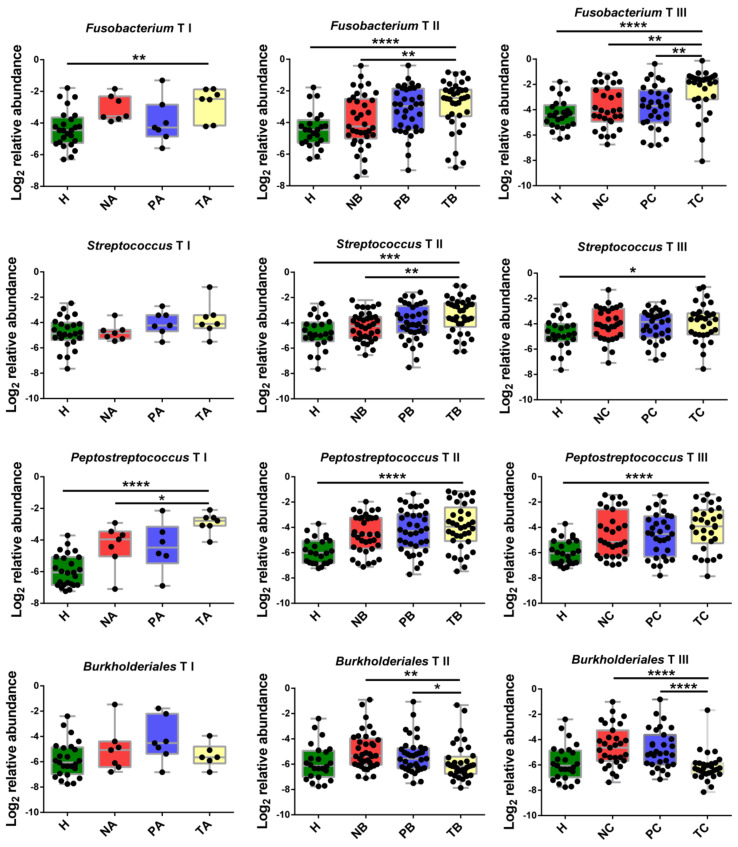
Abundance change analysis of CRC stage-specific biomarkers including *Fusobacterium*, *Streptococcus*, *Peptostreptococcus* and *Burkholderiales*. A: CRC stage I; B: CRC stage II; C: CRC stage III. N: off-tumor site; P: adjacent-tumor site; T: on-tumor site. H: healthy control. Abundance change differences were compared using one-way ANOVA with Kruskal–Wallis test, *, *p* < 0.05; **, *p* < 0.1; ***, *p* < 0.01; ****, *p* < 0.001.

**Figure 7:**
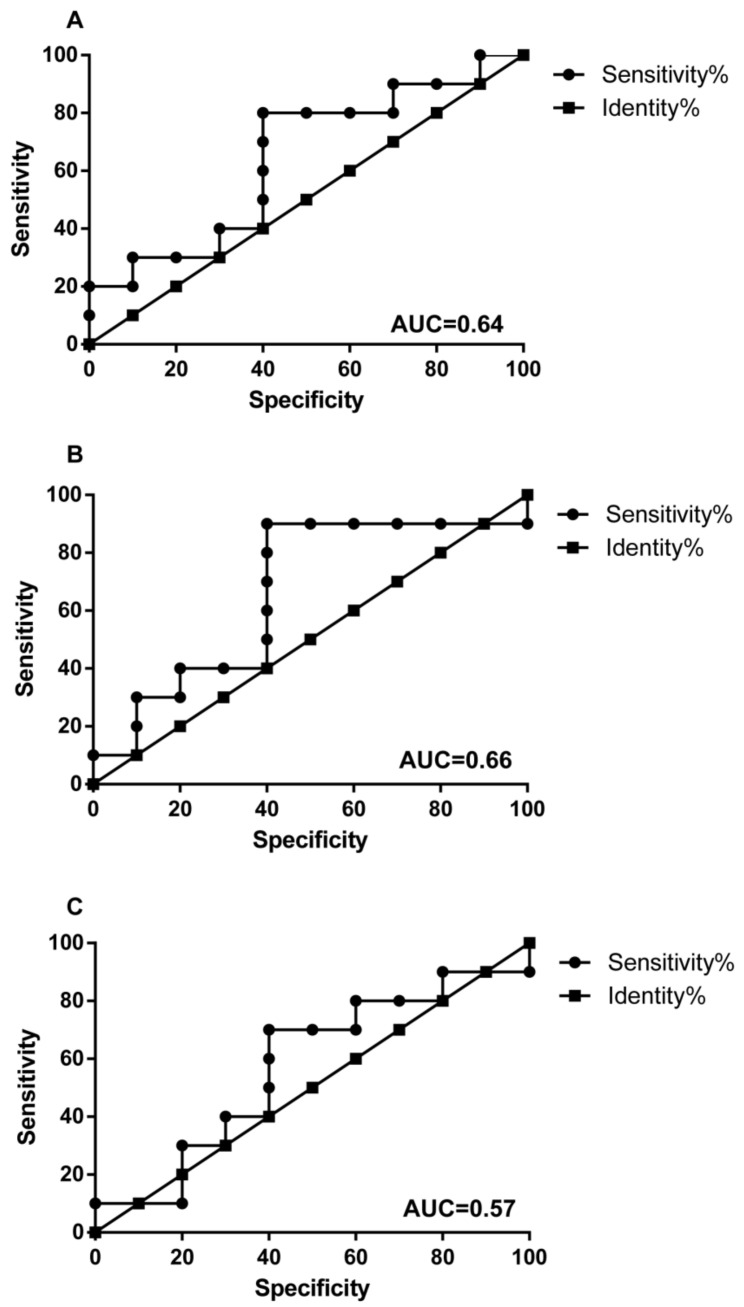
Receiver operating characteristic curve analysis showing diagnostic performance of *Fusobacterium*, *Peptostreptococcus*, *Streptococcus*, *Parvimonas*, *Burkholderiales*, *Caulobacteraceae*, Delftia, *Faecalibacterium*, *Oxalobacteraceae* and *Sutterella* to differentiate stage I and II (**A**), I and III (**B**), and II and III (**C**). AUC are above 0.5. AUC: areas under the receiver-operating curve.

As the microbial abundance patterns in T-site varied among CRC stages, we next aimed to identify CRC-stage specific potential markers at genus level to facilitate CRC-stage diagnose. To avoid variation noise of microbiome structure caused by individual difference, the optimized method is comparing the microbial abundances in T-site to those in P- or N-sites instead of healthy controls. Using this method, we identified 10 genera or families with abundance > 1% that showed significant differences in abundance between T-site and P/N-site in certain CRC stage (Figure 6, Figure S1). *Fusobacterium* in T-site showed significantly higher abundances than those in N-site at CRC stage II and those in P- and N-tumor sites at CRC stage III (Figure 6). However, at CRC stage I, the abundances of *Fusobacterium* in the three sites were not distinguishable (Figure 6). Additionally, *Peptostreptococcus*, *Parvimonas* and *Streptococcus* in T-site showed increased abundances and can serve as biomarkers for stages I, I-II and II, respectively (Figure 6, Figure S1). Other six genera/families with unknown genus showed decreased abundances in T-site compared to P/N-sites. *Burkholderiales*, *Caulobacteraceae*, *Oxalobacteraceae* can serve as biomarkers for CRC stages II and III (Figure 6, Figure S1). *Delftia* can serve as biomarker for CRC stage II, while *Faecalibacterium* and *Sutterella* can serve as biomarkers for CRC stage III (Figure S1). Using these ten genera/families with unknown genus as biomarkers, we performed receiver-operating characteristic analyses and CRC Stages I, II and III were distinguished from each other (Figure 7).

### 3.4. Oral Microbes Compared to Gut Mucosal Microbes in CRC Stages

**Figure 8:**
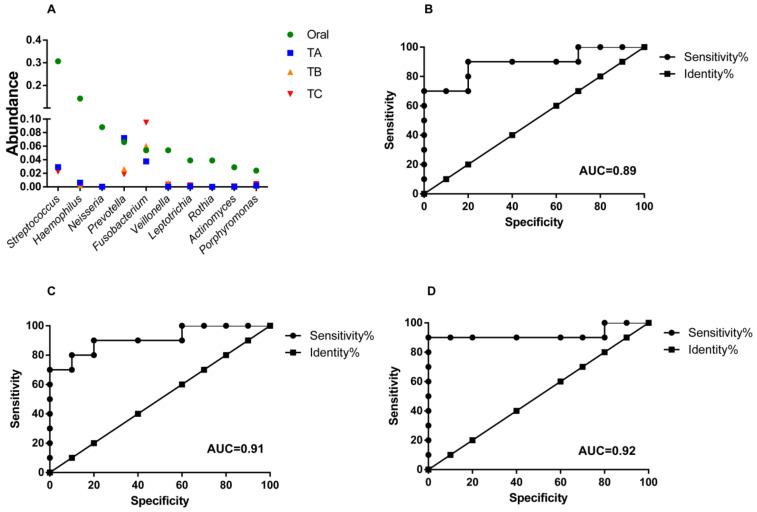
Abundance comparison of the top abundant oral bacteria among those in on-tumor sites at CRC stages I, II and III. (**A**). Abundance change comparison. (**B**–**D**) Receiver operating characteristic curve analysis showing diagnostic performance of the top abundant oral bacteria, to differentiate CRC stage I and oral cavity (**B**), II and oral cavity (**C**), and III and oral cavity (**D**). Areas under the receiver-operating curve are 0.89 (**B**), 0.91 (**C**) and 0.92 (**D**). A: CRC stage I; B: CRC stage II; C: CRC stage III. N: off-tumor site; P: adjacent-tumor site; T: on-tumor site. H: healthy control. AUC: areas under the receiver-operating curve.

Oral bacteria are able to cross the gastric mucosal barrier to enter and colonize in the colon [28]. We next examined whether the most abundant oral bacteria from individuals with CRC were present in T-site at CRC stages. Among the top 10 abundant oral bacteria, only *Streptococcus*, *Prevotella* and *Fusobacterium* were present in T-site with abundances >2% (Figure 8A). Only *Fusobacterium* in T-site at CRC stage III showed significantly higher abundance than that in oral cavity, whereas *Fusobacterium* at CRC stage II showed similar abundance to that in oral cavity (Figure 8A). Moreover, *Fusobacterium* in T-site at CRC stage I showed lower abundance than that in oral cavity (Figure 8A). Using these top abundant oral genera as markers, we performed receiver-operating characteristic analyses and CRC stages I, II and III were distinguished from oral cavity (Figure 8B–D).

### 3.5. Co-Occurrence and Co-Excluding Alteration of Gut Microbes during CRC Development

**Figure 9:**
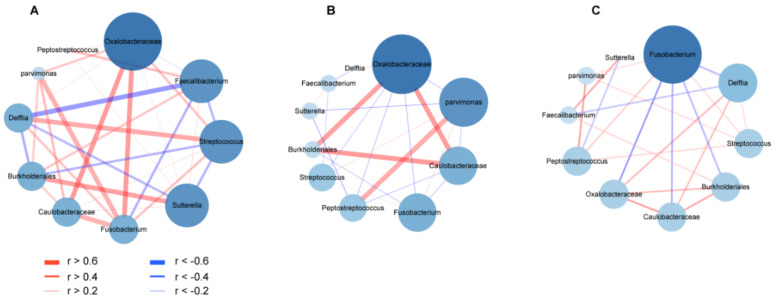
Co-occurrence and co-excluding interaction networks of CRC stage-specific markers showing profiles of microbiome in on-tumor site during CRC progression. (**A**). Co-occurrence and co-excluding interaction networks at CRC stage I. (**B**). Co-occurrence and co-excluding interaction networks at CRC stage II. (**C**). Co-occurrence and co-excluding interaction networks at CRC stage III. Bacteria pairs with Spearman rank correlation >0.2 or <−0.2 are represented in the network. Edges represent co-occurrence or co-excluding interaction relationships between bacteria pairs. Cytoscape v3.7.2 was used for co-occurrence and co-excluding interaction network construction. The size of the nodes corresponds to edge degree.

As bacterial co-occurrence and co-excluding interactions in mixed-species populations are essential to reveal bacterial competitions and maintenance of community composition, we next examined co-occurrence and co-excluding interaction networks among pairwise bacterial markers for CRC-specific stages in T-, P- and N-sites during CRC progression. In T-site, bacteria exhibited more interaction relationships and higher Spearman rank correlation values at CRC stage I than those at CRC stages II and III (Figure 9), which was also observed when more genera (abundance > 0.1%) were analyzed (Figure S2), suggesting profiles of gut mucosal microbiomes occurred during CRC progression. In P- and N-sites, bacteria showed similar relationship patterns to those in the T-site (Figure S3). These results were consistent with the above observation that the abundances of four biomarkers increased and those of other bacterial markers went down during CRC progression. Thus, certain factors from intestinal mucosal microenvironment shared among the T-, N- and P-sites lead to the same interaction pattern in the survival of coexisting species and depletion of excluding species. At CRC stage I, *Fusobacterium* served as the scaffolding bacterium that positively correlated with the highest number of bacteria (r > 0.4, edge degree = 5) in T-site, including *Parvimons*, *Caulobacteraceae* and *Oxalobacteraceae* with strongest strengths (r > 0.6), as well as *Delftia* and *Streptococcus* with less strong relationships (0.6 > r > 0.4) (Figure 9A). However, these strong co-occurrence relationships lost at CRC stages II and III (r > 0.4) (Figure 9B,C). In T-site, *Fusobacterium* showed weak positive relationships with *Parvimonas* and *Streptococcus* (0.4 > r > 0.2) at CRC stage II, and with *Streptococcus* and *Peptostreptococcus* (0.4 > r > 0.2) at CRC stage III (Figure 9B,C). At CRC stage I, *Fusobacterium* showed strong positive relationships with *Peptostreptococcus*, *Parvimonas*, *Caulobacteraceae*, *Oxalobacteraceae* and *Burkholderiales* in N-site (r > 0.4), and with *Sutterella*, *Caulobacteraceae* and *Burkholderiales* in P-site (r > 0.6) (Figure S3). Consistently, *Fusobacterium* only showed weak positive relationships with *Parvimonas*, *Peptostreptococcus* and *Streptococcus* in N- and P-sites at CRC stages II and III (0.4 > r > 0.2) (Figure S3).

## 4. Discussion

Early detection and treatment of CRC can improve survival rates for patients, e.g., >90% surviving at 5 years for patients diagnosed with stage I versus 50% surviving at 5 years for those with stage III [29,30]. Thus, discovering new bacterial biomarkers associated with specific CRC stages will facilitate accurate diagnosis of CRC and staging. On the other hand, studies on the functional roles of these pathogenic bacteria in CRC development will help develop feasible treatment of CRC and prevent its recurrence. In this work, to avoid the noise brought by inter-individual variations in gut mucosal microbiome, we analyzed gut mucosal microbiomes of triplet-paired biopsy samples collected from on-tumor (T), adjacent-tumor (P) and off-tumor (N) sites of CRC patients to identify potential biomarkers for CRC stages. Our results establish a set of bacterial taxa as potential biomarkers specific for CRC stages I, II and III. PCA analysis of composition structures of gut mucosal microbiomes showed differences in T-, P- and N-sites at the same stage, as well as in the same site as stage changes, suggesting that there are differential abundances of bacterial genera associated with specific sites as CRC develops. We identified 10 bacterial genera/families with unknown genus with top abundances (>1%) as biomarkers for CRC stages, including *Peptostreptococcus* (stage I), *Parvimonas* (stages I and II), *Streptococcus* (stage II), *Delftia* (stage II), *Fusobacterium* (stages II and III), *Burkholderiales* (stages II and III), *Caulobacteraceae* (stages II and III), *Oxalobacteraceae* (stages II and III), *Faecalibacterium* (stage III) and *Sutterella* (stage III). Our analyses of co-occurrence and co-excluding interaction networks further showed a loss-of-interaction pattern in T-site as well as in N- and P-sites during CRC development, indicating TME-associated microbial profiles occur differentially in CRC stages.

The CRC-stage specific biomarkers characterized in this work reflect that *Peptostreptococcus*, instead of notorious human pathogen *Fusobacterium*, showed significantly differential abundances between T- and N-sites at CRC stage I, although both of them showed higher abundances in T-site than those in healthy controls at CRC stage I. At CRC stages II and III, *Peptostreptococcus* only showed higher abundances in T-site than healthy controls, whereas *Fusobacterium* showed significantly differential abundances between T- and N-sites/healthy controls. These data indicate that *Peptostreptococcus* serves as the major driver for tumor progression at early-stage CRC, and its role is gradually replaced by *Fusobacterium* and others at late-stage CRC. The oncogenic potential of *Peptostreptococcus anaeerobius* has been investigated using a mouse model, which confirmed that *P. anaeerobius* is able to promote proliferation of colonic epithelial cells and modulate the immune microenvironment [5]. *Fusobacterium nucleatum*, well recognized as a key pathogen in gingivitis and periodontitis, has recently drawn attentions on its role in CRC tumorigenesis and metastasis [31,32,33,34,35,36]. Our findings indicate that a well-established TME is more suitable for *Fusobacterium* proliferation that further modulates proinflammatory TME for metastasis, whereas the role of *Peptostreptococcus* may be masked by the highly abundant *Fusobacterium* (10% vs. 2% *Peptostreptococcus*, Figure 3A) in late-stage CRC.

Additionally, *Parvimonas* (stages I and II) and *Streptococcus* (stage II) with top abundances in gut mucosal microbiome were identified as biomarkers for specific CRC stages in this study. *Parvimonas micros* is commonly found in the commensal flora of the gingival crevice and frequently isolated in polymicrobial periodontitis [37]. Recent evidence suggests that *P. micros* significantly promotes proliferation of colon cell lines NCM460, HT-29 and Caco-2 and enhances gut inflammation [38]. *Sgg* relies on its type VII secretion systems to promote its adherence to HT29 cells and stimulate HT29 cell proliferation [39]. Moreover, *Sgg* was shown to prefer TME for colonization at the expense of resident intestinal enterococci, by secreting an active bacteriocin in bile acids [40]. Thus, *Parvimonas* and *Streptococcus* are involved in establishing TME in CRC stages I-II and II.

The remaining CRC stage-specific biomarkers including, *Burkholderiales*, *Caulobacteraceae*, *Oxalobacteraceae*, *Delftia*, *Faecalibacterium* and *Sutterella*, showed lower abundances in T-site than N- or P- sites. As their abundances and tumor progression are negatively correlated, their roles associated with gut microbiome profiles and tumorigenesis are less well characterized. A few studies suggest that *Faecalibacterium* plays a major role in the regulation of gut barrier, inflammation and metabolic functions [41]. *Sutterella*, associated with gastrointestinal diseases, does not induce substantial inflammation but has a capacity to degrade IgA [42]. Further characterization of the functional roles of these genera in TME and their excluding interactions with *Fusobacterium* at late stage of CRC that involve interspecies competitions will help understand the factors that result in their depletion in the community.

The bacterial density in the colon reaches extremely high level, viz 10^11^ bacterial cells per 1 mL colon content [43], indicating that gut mucosal microbiota may form biofilm-like structure and bacteria physically interact with each other. Bacterial co-occurring and co-excluding interactions in the networks of mixed-species populations may reflect CRC stage-specific TME statuses and serve as a CRC stage-specific indicator. We identified a loss-of-interaction pattern for CRC progression in the T-, P- and N-sites, indicating the profiles of gut microbiota severed as CRC evolves. *Fusobacterium* maintained a weak co-occurrence interaction with *Peptostreptococcus*, *Streptococcus* and *Parvimonas* in the T-, P- and N-sites at CRC stage III. *F. nucleatum* functions as a bridge-forming bacterium to interact with other bacterial colonizers, leading to most complex biofilm formation in the human body [44,45]. It is postulated that *Fusobacterium* in the colon of CRC patients comes from the oral cavity due to its absence in healthy colon and its abundance in human oral cavity [46]. However, because our sequencing depth is deep enough, we find *Fusobacterium* in healthy controls, revealing that this genus resides in healthy colon with low abundance instead of translocation from the oral cavity through gastric mucosal barrier or blood at any stage of CRC. Our data further reveal that the abundance of *Fusobacterium* in T-site at CRC stage III but not in other conditions is higher than those in oral cavity with CRC (Figure 3A). However, the absolute total number of bacteria in colon is higher than that in oral cavity (10^11^ CFU/mL in colon vs 10^7^ CFU/mL in oral cavity) [47,48]. Therefore, the absolute number of *Fusobacterium* in colon is higher than that in oral cavity. Since the compositions of both bacterial communities and eukaryotic cells in colon and oral cavity are respectively distinct, *Fusobacterium* may play different roles in colon and oral cavity. The TME at CRC stage III may favor *F. nucleatum* proliferation that functions as a bridge to form multi-species biofilm containing pathogenic *Peptostreptococcus*, *Streptococcus* and *Parvimonas*. However, these bacteria thrive at the expense of other species.

Our analyses showed a gradual switch of microbial profiles during CRC progression and classified bacterial taxa to four groups (group I: stage I > II > III; group II: stage I < II < III; group III: stage I > II < III; group IV: stage I < II > III) (Figure 4A–D, Table S4). The group I bacteria gradually lose growth advantage as CRC develops and TME changes, suggesting these bacteria may function as either probiotics (e.g., *Bacteroides* and *Ruminococcus*) or pathogenic bacteria (e.g., *Prevotella* and *Eubacterium*) adapted to early TME (Table S4). Their proliferation abilities are outcompeted by group II bacteria that gradually increase the relative abundances as TME changes. Besides *Fusobacterium*, group II bacteria contain several famous pathogenic genera in human infectious diseases, such as *Klebsiella*, *Pseudomonas*, *Vibrio* and *Mycobacterium* that produce known virulence factors in response to host immune systems (Table S4). These bacteria show highest proliferation abilities in CRC stage III with the most complex TME containing immune cells with migratory capacities such as macrophages, neutrophils and fibroblasts. Their pathogenicity may play roles in shaping the late TME and involving metastases. Intriguingly, group III and IV bacteria show the lowest and highest abundances in CRC stage II, respectively, indicating that the complex TME in stage III benefits the proliferation of group III bacteria but impairs group IV bacterial growth. Group IV bacteria contain several famous pathogenic genera in human infectious diseases such as *Streptococcus*, *Campylobacter* and *Clostridium* (Table S4). These bacteria may drive early CRC development but gradually lose adaptation to TME in CRC stage III.

Our study defines the gut mucosal community in stage-specific CRC for tumorigenesis. We identified CRC stage-specific biomarkers that have potentials for clinical diagnosis and showed profiles of gut mucosal microbiome as CRC progresses. In future work, collection of larger sizes of samples along with experimental validation will help to provide a more accurate profile of CRC-stage associated biomarkers. In addition, further functional analyses of interplay between gut microbes and host immune cells will help understand the roles of gut microbiomes in human diseases.

## Data Availability

The 16S rRNA sequencing reads have been submitted to the NCBI SRA database under accession number PRJNA606879. All the data are provided in this manuscript and Supplementary Materials.

## Supplementary Materials

The following are available online at https://www.mdpi.com/article/10.3390/life11080831/s1, Figure S1: Abundance change analysis of CRC stage-specific biomarkers. Figure S2: Co-occurrence and co-excluding interaction networks of genera with abundance >0.1% showing profiles of microbiome in on-tumor site during CRC progression. Figure S3: Co-occurrence and co-excluding interaction networks of CRC stage-specific markers showing profiles of microbiome in adjacent-tumor site and off-tumor site during CRC progression. Table S1: Summary of CRC patients and healthy people. Table S2: Summary of assembled reads numbers. Table S3: List of genera/families with unknown genus shared among CRC stages I, II and III at on-tumor site (T) and healthy controls. Table S4: Summary of bacterial taxa identified in four groups shown in Figure 4.
