# Supplementary material for: Differential Mucosal Microbiome Profiles across Stages of Human Colorectal Cancer
Source: Life (Basel). 2021 Aug 13;11(8):831. doi: 10.3390/life11080831 (PMC8401903; doi:10.3390/life11080831)
Supplement: Supplementary file 1 [file life-11-00831-s001.zip › Supplementary Figures.pdf]

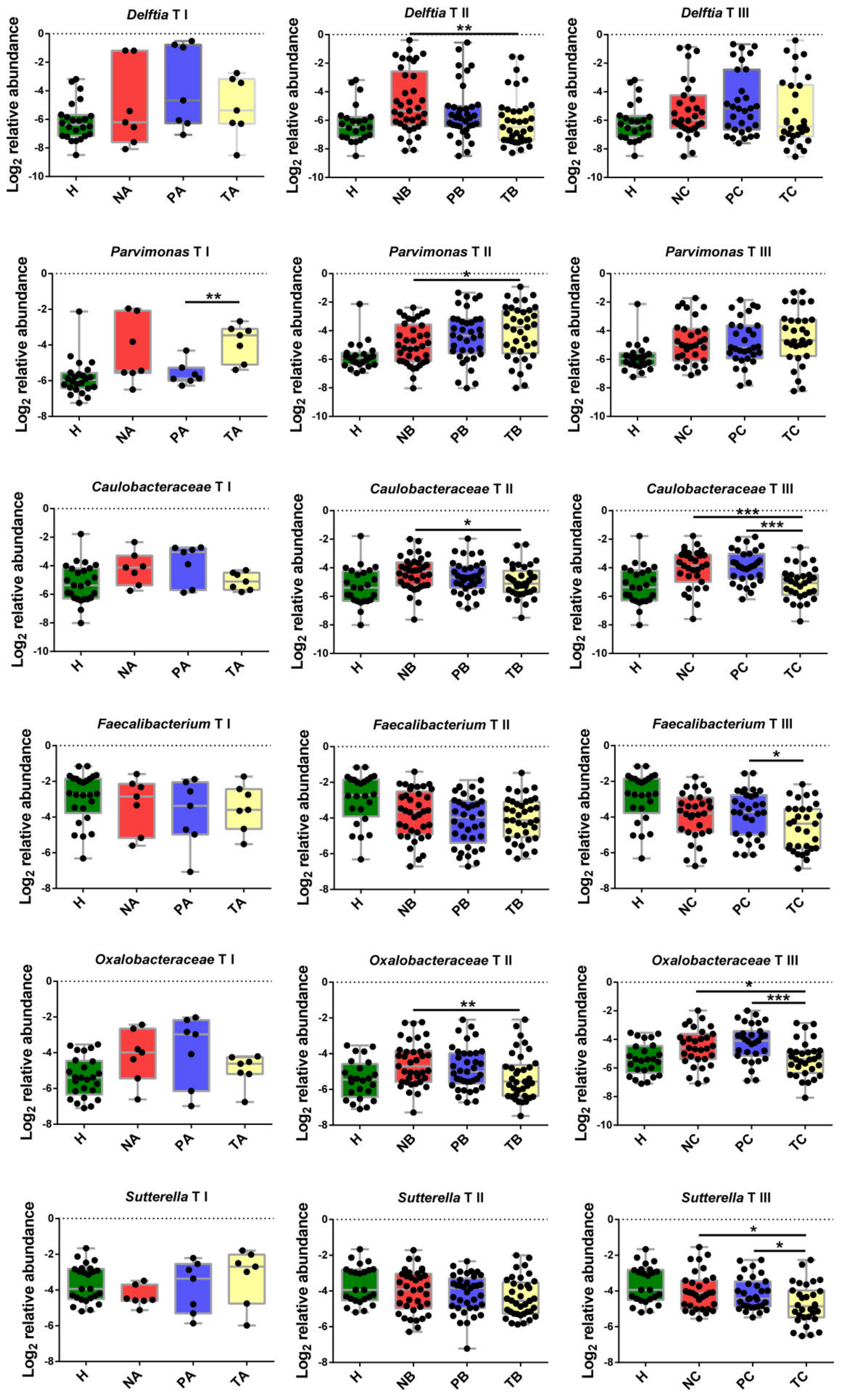

Scheme 1. Abundance change analysis of CRC stage-specific biomarkers. A: CRC stage I; B: CRC stage II; C: CRC stage III. Abundance change differences were compared using one-way ANOVA with Kruskal–Wallis test, *, $p<0.05$; **, $p<0.1$; ***, $p<0.01$.

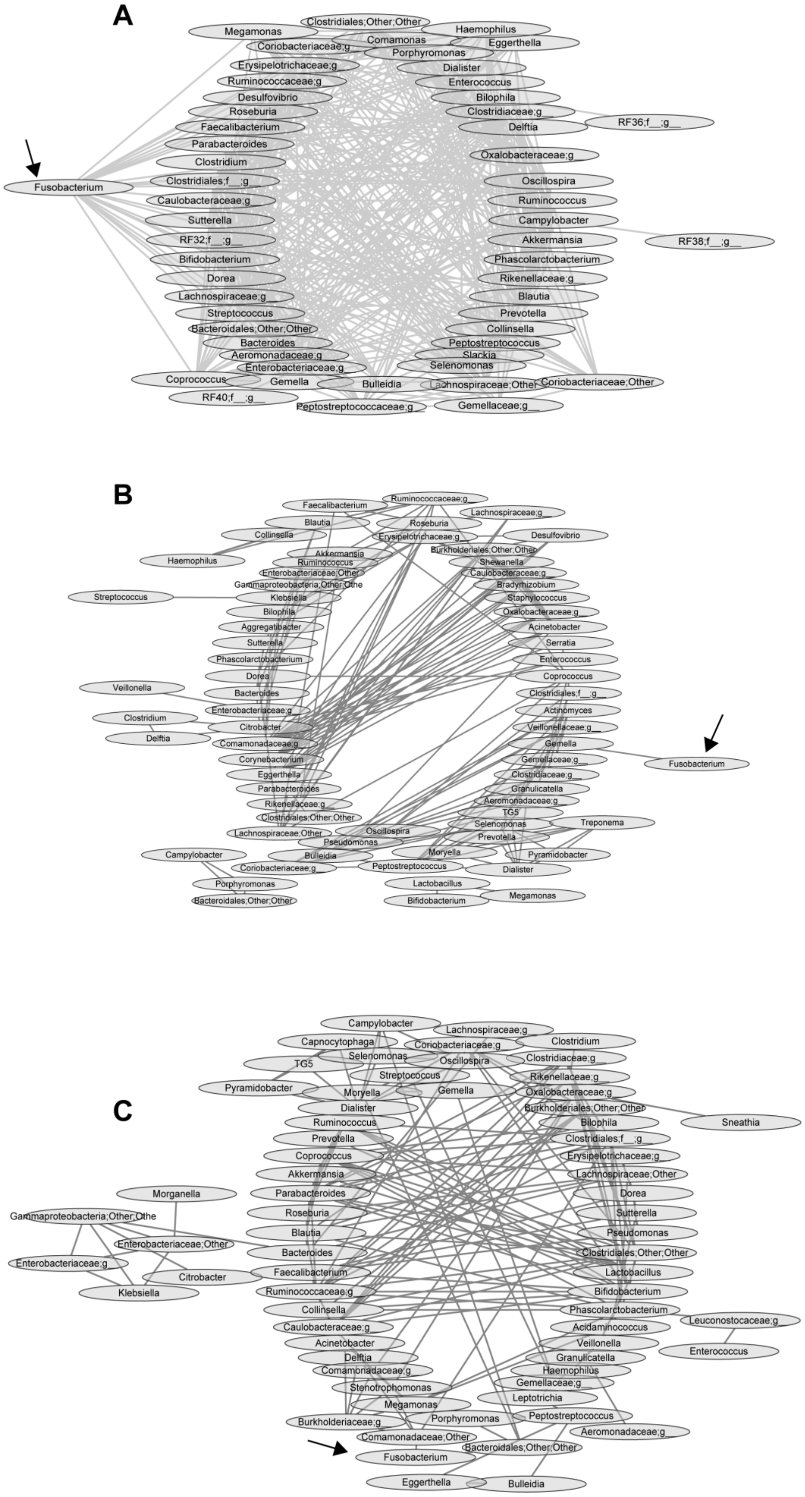

Scheme 2. Co-occurrence and co-excluding interaction networks of genera with abundance > 0.1% showing profiles of microbiome in on-tumor site during CRC progression. (A). Co-occurrence and co-excluding interaction networks at CRC stage I. (B). Co-occurrence and co-excluding interaction networks at CRC stage II. (C). Co-occurrence and co-excluding interaction networks at CRC stage III. Bacteria pairs with Spearman rank correlation > 0.4 or < -0.4 are represented in the network. Edges represent co-occurrence or co-excluding interaction relationships between bacteria pairs. Cytoscape v3.7.2 was used for co-occurrence and co-excluding interaction network construction. Black arrow indicates *Fusobacterium*.

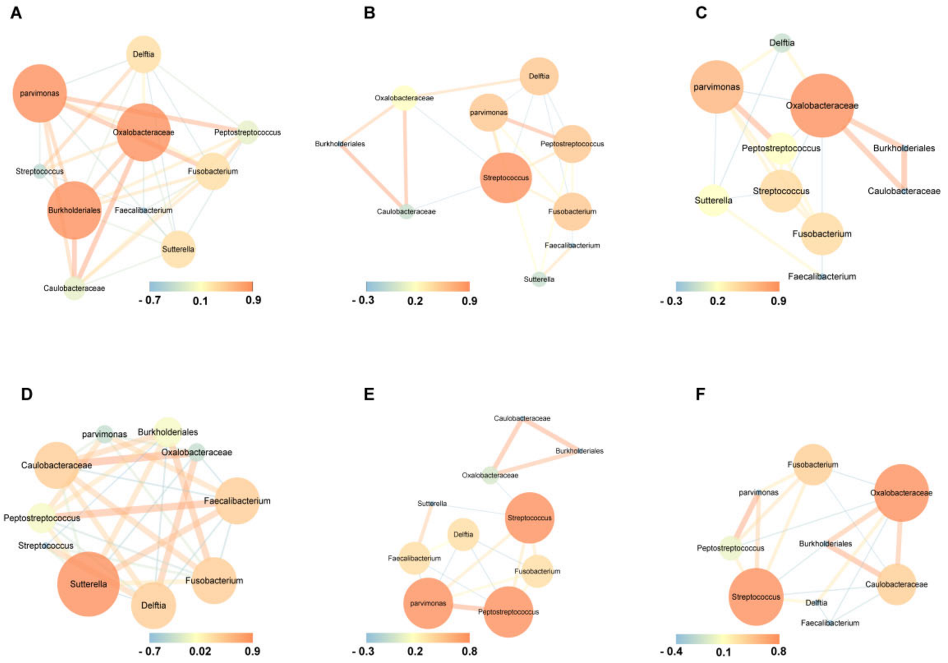

Scheme 3. Co-occurrence and co-excluding interaction networks of CRC stage-specific markers showing profiles of microbiome in adjacent-tumor site and off-tumor site during CRC progression. (A). Co-occurrence and co-excluding interaction networks in adjacent-tumor site at CRC stage I. (B). Co-occurrence and co-excluding interaction networks in adjacent-tumor site at CRC stage II. (C). Co-occurrence and co-excluding interaction networks in adjacent-tumor site at CRC stage III. (D). Co-occurrence and co-excluding interaction networks in off-tumor site at CRC stage I. (E). Co-occurrence and co-excluding interaction networks in off-tumor site at CRC stage II. (F). Co-occurrence and co-excluding interaction networks in off-tumor site at CRC stage III. Bacteria pairs with Spearman rank correlation > 0.2 or < -0.2 are represented in the network. Edges represent co-occurrence or co-excluding interaction relationships between bacteria pairs. Cytoscape v3.7.2 was used for co-occurrence and co-excluding interaction network construction. The size of the nodes corresponds to edge degree.
