# Supplementary material for: Differential Mucosal Microbiome Profiles across Stages of Human Colorectal Cancer
Source: Life (Basel). 2021 Aug 13;11(8):831. doi: 10.3390/life11080831 (PMC8401903; doi:10.3390/life11080831)
Supplement: Supplementary file 1 [file life-11-00831-s001.zip › Supplementary Table 1.pdf]

**Supplementary table 1. Summary of CRC patients and healthy people.** The numbers correspond to the numbers in the sample names of 16S rRNA sequencing reads (supplementary table 2).

CRC patients diagnosed with stage I

| number | sex | age | location | type (adenocarcinoma) | TNM | pathological diagnostic image |
|---|---|---|---|---|---|---|
| 5 | female | 71 | colon | moderate differentiated | $PT_2N_0M_x$ | 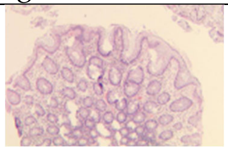 |
| 9 | male | 78 | rectum | moderate differentiated | $PT_2N_0M_x$ | 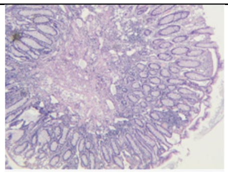 |
| 11 | male | 56 | rectum | moderate differentiated | $PT_2N_0M_x$ | 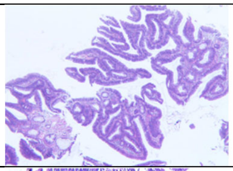 |
| 38 | female | 74 | colon | moderate differentiated | $PT_2N_0M_x$ | 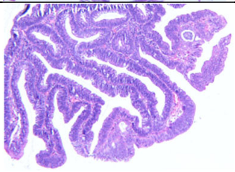 |
| 41 | female | 65 | colon | moderate differentiated | $PT_2N_0M_x$ | 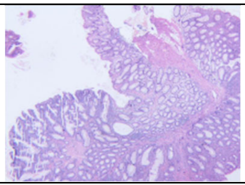 |

| | | | | | | |
|---|---|---|---|---|---|---|
| 66 | male | 73 | rectum | well/moderate differentiated | $PT_2N_0M_X$ | 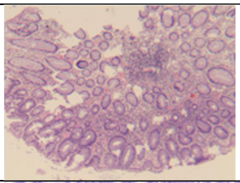 |
| 74 | female | 64 | colon | moderate differentiated | $PT_2N_0M_X$ | 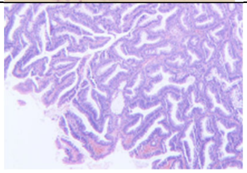 |

CRC patients diagnosed with stage II

| number | sex | age | location | type (adenocarcinoma) | TNM | pathological diagnostic image |
|---|---|---|---|---|---|---|
| 6 | female | 61 | rectum | moderate differentiated | $PT_3N_0M_X$ | 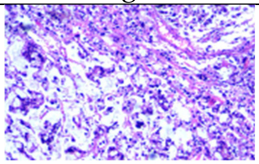 |
| 12 | male | 66 | rectum | poorly differentiated | $PT_3N_0M_X$ | 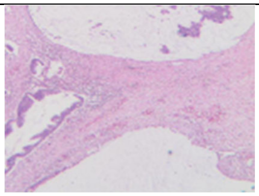 |
| 13 | male | 70 | rectum | moderate differentiated | $PT_3N_0M_X$ | 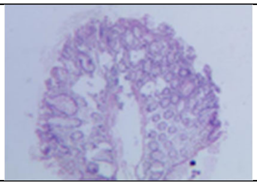 |

| | | | | | | |
|---|---|---|---|---|---|---|
| 17 | male | 50 | rectum | moderate differentiated | $PT_3N_0M_X$ | 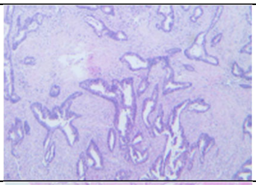 |
| 18 | male | 72 | rectum | moderate differentiated | $PT_3N_0M_X$ | 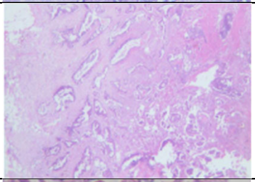 |
| 25 | male | 64 | rectum | moderate/poorly differentiated | $PT_3N_0M_X$ | 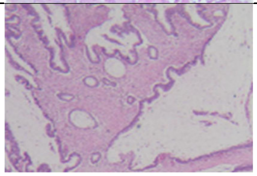 |
| 28 | male | 67 | rectum | poorly differentiated | $PT_3N_0M_X$ | 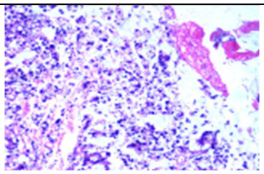 |
| 30 | male | 71 | rectum | mucinous | $PT_3N_0M_X$ | 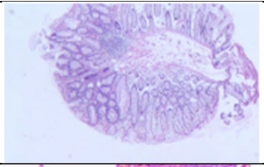 |
| 37 | male | 52 | rectum | moderate differentiated | $PT_3N_0M_X$ | 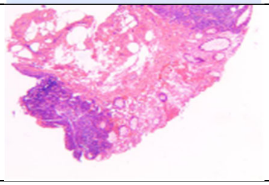 |

| | | | | | | |
|---|---|---|---|---|---|---|
| 43 | male | 68 | rectum | moderate differentiated | $PT_3N_0M_X$ | 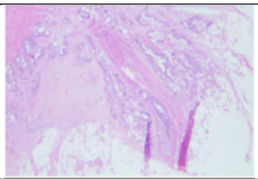 |
| 48 | male | 62 | rectum | moderate/poorly differentiated | $PT_3N_0M_X$ | 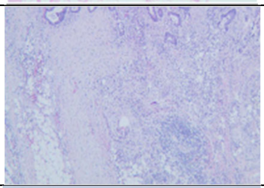 |
| 49 | male | 61 | rectum | moderate differentiated | $PT_3N_0M_X$ | 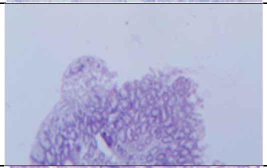 |
| 50 | female | 81 | rectum | moderate differentiated | $PT_4N_0M_X$ | 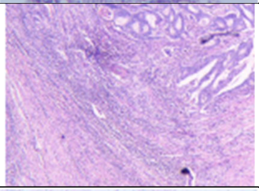 |
| 51 | male | 71 | rectum | moderate differentiated | $PT_3N_0M_X$ | 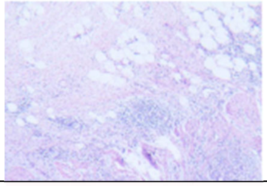 |
| 52 | male | 61 | rectum | moderate differentiated | $PT_3N_0M_X$ | 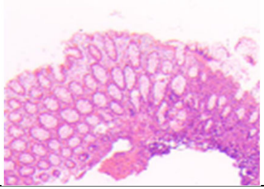 |

| | | | | | | |
|---|---|---|---|---|---|---|
| 57 | male | 60 | rectum | moderate differentiated | $PT_3N_0M_X$ | 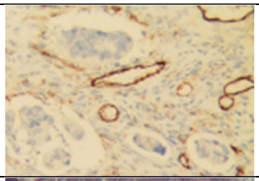 |
| 58 | male | 66 | rectum | moderate differentiated | $PT_3N_0M_X$ | 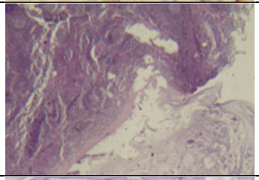 |
| 60 | male | 81 | rectum | moderate differentiated | $PT_3N_0M_X$ | 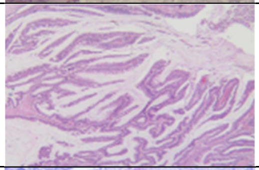 |
| 61 | male | 55 | rectum | moderate differentiated | $PT_3N_0M_X$ | 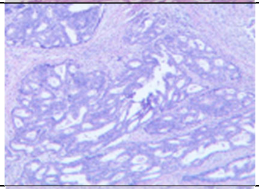 |
| 62 | female | 75 | rectum | moderate differentiated | $PT_3N_0M_X$ | 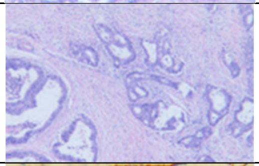 |
| 65 | female | 74 | rectum | moderate differentiated | $PT_3N_0M_X$ | 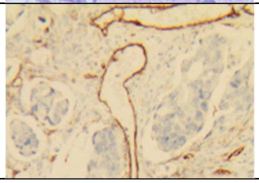 |

| | | | | | | |
|---|---|---|---|---|---|---|
| 69 | female | 43 | rectum | moderate differentiated | $PT_3N_0M_x$ | 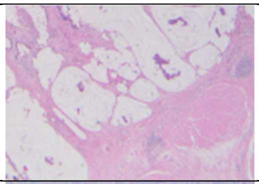 |
| 71 | female | 76 | rectum | moderate differentiated | $PT_3N_0M_x$ | 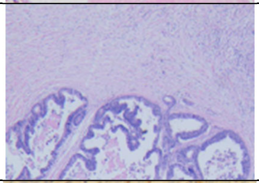 |
| 1 | male | 76 | sigmoid colon | well/moderate differentiated | $PT_3N_0M_x$ | 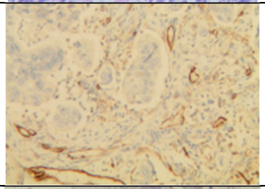 |
| 8 | female | 57 | right colon | well/moderate differentiated | $PT_3N_0M_x$ | 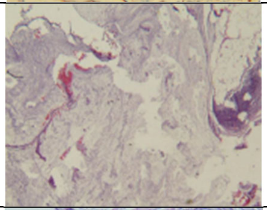 |
| 14 | female | 68 | right colon | mucinous | $PT_3N_0M_x$ | 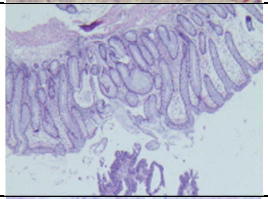 |
| 21 | female | 65 | sigmoid colon | moderate differentiated | $PT_3N_0M_x$ | 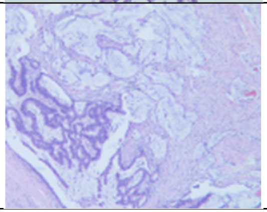 |

| | | | | | | |
|---|---|---|---|---|---|---|
| 22 | female | 48 | colon | moderate differentiated | $PT_3N_0M_X$ | 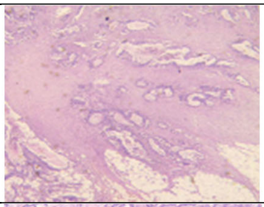 |
| 24 | female | 76 | colon | moderate differentiated | $PT_3N_0M_X$ | 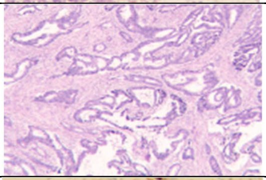 |
| 27 | male | 53 | rectosigmoid colon | rectumcancer | $PT_3N_0M_X$ | 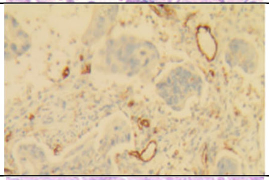 |
| 44 | male | 64 | sigmoid co-lon | moderate-poorly differenti-ated | $PT_4N_0M_X$ | 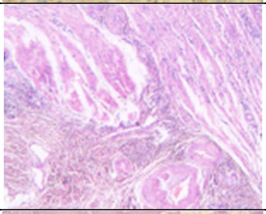 |
| 45 | male | 74 | sigmoid co-lon | moderate differentiated | $PT_3N_0M_X$ | 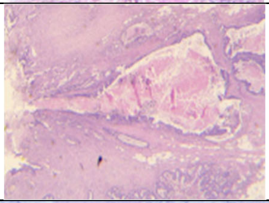 |
| 54 | male | 59 | sigmoid co-lon | moderate-poorly differenti-ated | $PT_3N_0M_X$ | 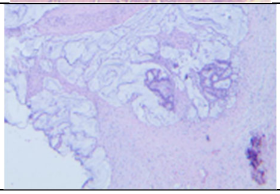 |

| | | | | | | |
|---|---|---|---|---|---|---|
| 55 | female | 45 | right colon | moderate-poorly differentiated | $PT_4N_0M_X$ | 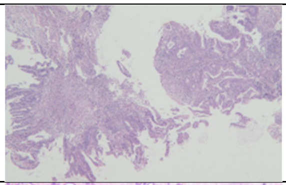 |
| 63 | female | 57 | rectosigmoid colon | moderate differentiated | $PT_4N_0M_X$ | 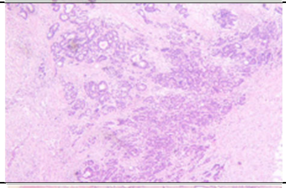 |
| 68 | male | 70 | rectosigmoid colon | moderate differentiated | $PT_4N_0M_X$ | 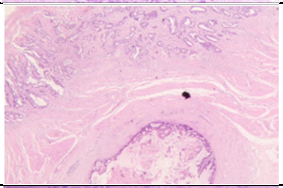 |
| 72 | female | 53 | left colon | moderate differentiated | $PT_4N_0M_X$ | 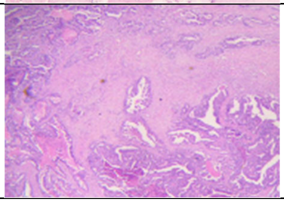 |

CRC patients diagnosed with stage III

| number | sex | age | location | type (adenocarcinoma) | TNM | pathological diagnostic image |
|---|---|---|---|---|---|---|
| 2 | male | 69 | rectum | moderate differentiated | $PT_4N_2M_X$ | 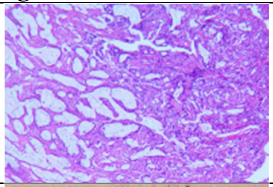 |
| 7 | female | 57 | rectum | moderate differentiated | $PT_3N_1M_X$ | 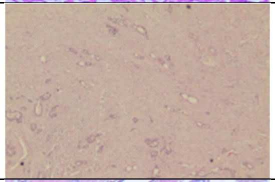 |
| 10 | female | 65 | rectum | moderate differentiated | $PT_3N_1M_X$ | 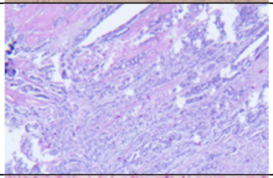 |
| 16 | female | 55 | rectum | poorly differentiated | $PT_3N_2M_X$ | 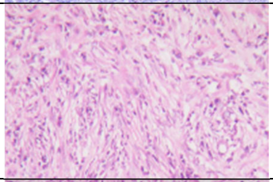 |
| 19 | male | 58 | rectum | moderate differentiated | $PT_3N_2M_X$ | 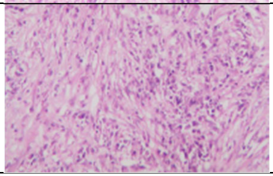 |

| | | | | | | |
|---|---|---|---|---|---|---|
| 20 | male | 60 | rectum | poorly differentiated | $PT_3N_2M_X$ | 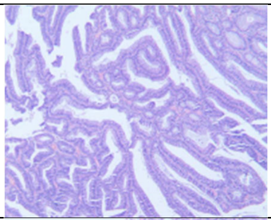 |
| 23 | male | 68 | rectum | poorly differentiated | $PT_3N_2M_X$ | 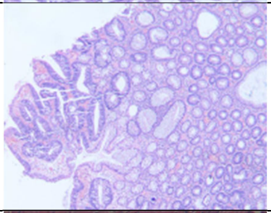 |
| 32 | female | 49 | rectum | moderate-poorly differentiated | $PT_2N_1M_X$ | 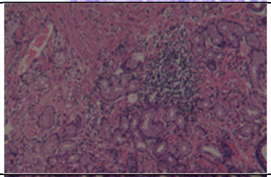 |
| 34 | female | 72 | rectum | moderate-poorly differentiated | $PT_2N_1M_X$ | 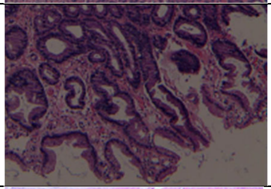 |
| 35 | male | 79 | rectum | moderate-poorly differentiated | $PT_4N_1M_X$ | 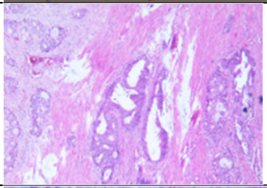 |
| 36 | male | 29 | rectum | moderate differentiated | $PT_4N_1M_X$ | 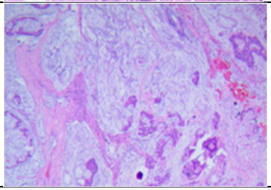 |

| | | | | | | |
|---|---|---|---|---|---|---|
| 40 | female | 61 | rectum | moderate differentiated | $PT_3N_1M_0$ | 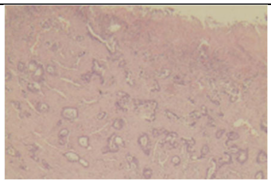 |
| 46 | female | 49 | rectum | moderate differentiated | $PT_3N_2M_X$ | 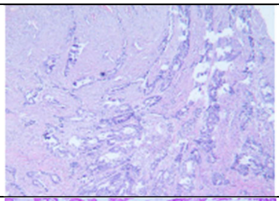 |
| 53 | male | 67 | rectum | moderate-poorly differentiated | $PT_4N_2M_X$ | 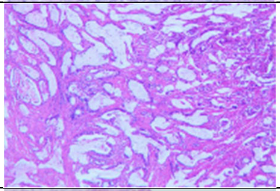 |
| 59 | female | 67 | rectum | moderate-poorly differentiated | $PT_4N_2M_X$ | 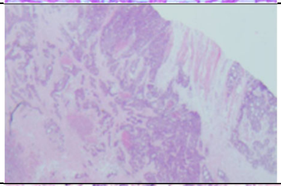 |
| 73 | male | 80 | rectum | poorly differentiated | $PT_4N_2M_X$ | 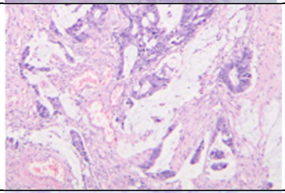 |
| 75 | male | 48 | rectum | poorly differentiated | $PT_4N_2M_X$ | 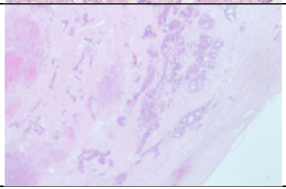 |

| | | | | | | |
|---|---|---|---|---|---|---|
| 3 | female | 68 | sigmoid colon | moderate differentiated | $PT_4N_1M_X$ | 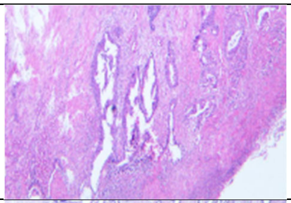 |
| 4 | male | 68 | sigmoid colon | moderate-poorly differentiated | $PT_4N_2M_X$ | 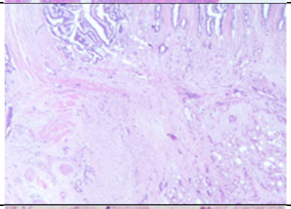 |
| 15 | male | 77 | colon | moderate-poorly differentiated | $PT_4N_2M_X$ | 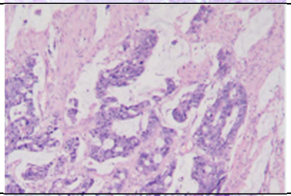 |
| 26 | female | 58 | rectosigmoid colon | moderate differentiated | $PT_4N_2M_X$ | 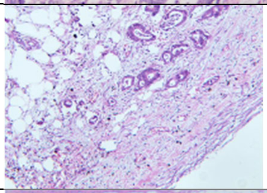 |
| 29 | male | 82 | rectosigmoid colon | moderate differentiated | $PT_3N_2M_X$ | 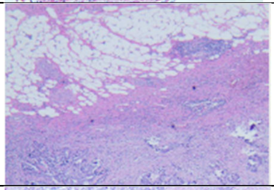 |
| 31 | male | 53 | colon | familial polyposis | $PT_3N_1M_X$ | 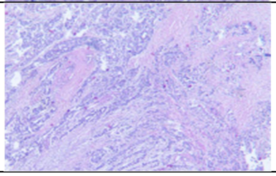 |

| | | | | | | |
|---|---|---|---|---|---|---|
| 56 | male | 60 | ileocecum lymphoma | moderate-poorly differentiated | $PT_3N_1M_X$ | 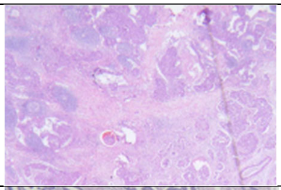 |
| 47 | female | 74 | right colon | moderate differentiated | $PT_3N_1M_X$ | 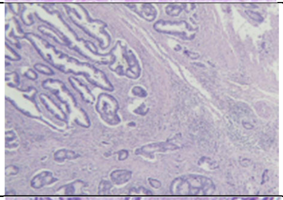 |
| 39 | female | 46 | right colon | moderate-poorly differentiated | $PT_4N_1M_X$ | 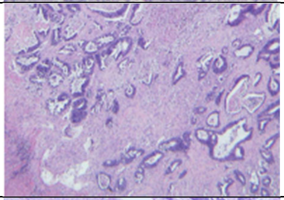 |
| 33 | male | 54 | rectosigmoid colon | rectumcancer | $PT_3N_2M_X$ | 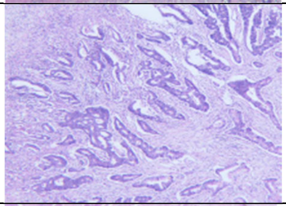 |
| 64 | female | 57 | rectosigmoid colon | moderate-poorly differentiated | $PT_3N_1M_X$ | 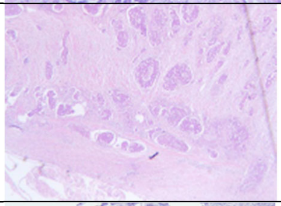 |
| 42 | male | 35 | descending colon | moderate differentiated | $PT_3N_1M_X$ | 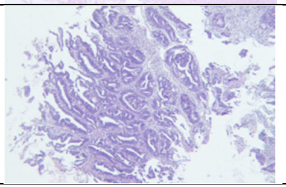 |

| | | | | | | |
|---|---|---|---|---|---|---|
| 67 | male | 58 | rectosigmoid colon | moderate differentiated | $PT_4N_1M_X$ | 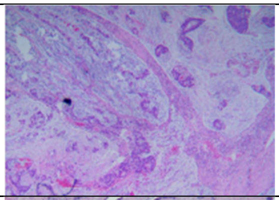 |
| 70 | male | 79 | rectosigmoid colon | mucinous | $PT_4N_1M_X$ | 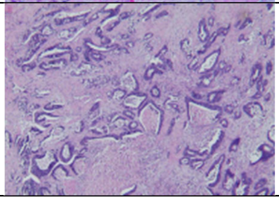 |
