# Supplementary material for: Differential Mucosal Microbiome Profiles across Stages of Human Colorectal Cancer
Source: Life (Basel). 2021 Aug 13;11(8):831. doi: 10.3390/life11080831 (PMC8401903; doi:10.3390/life11080831)
Supplement: Supplementary file 1 [file life-11-00831-s001.zip › Supplementary Table 2.pdf]

Supplementary table 2. Summary of assembled reads numbers.

Sample	reads	Sample	reads	Sample	reads	Sample	reads
N1	158681	P1	146450	T1	125279	H1	228226
N2	155520	P2	144239	T2	161009	H2	86770
N3	151493	P3	144342	T3	144093	H3	97081
N4	107871	P4	143790	T4	138683	H4	76774
N5	149236	P5	120410	T5	144144	H5	67552
N6	157024	P6	133251	T6	146166	H6	84126
N7	163467	P7	120907	T7	143849	H7	91269
N8	88856	P8	70370	T8	176639	H8	53616
N9	172902	P9	157359	T9	158859	H9	73912
N10	159768	P10	42129	T10	127105	H10	131958
N11	197144	P11	170524	T11	162382	H11	67801
N12	163288	P12	149258	T12	154349	H12	69007
N13	112378	P13	159419	T13	142627	H13	169592
N14	136611	P14	131512	T14	141903	H14	190398
N15	146468	P15	114142	T15	140699	H15	80341
N16	158784	P16	162133	T16	164102	H16	75367
N17	139945	P17	149499	T17	156325	H17	86805
N18	135714	P18	120591	T18	140324	H18	113854
N19	143846	P19	157367	T19	42985	H19	147101
N20	145935	P20	136699	T20	150887	H20	179992
N21	139062	P21	171269	T21	165212	H21	184803
N22	69623	P22	68371	T22	67025	H22	374772
N23	137444	P23	126077	T23	133157	H23	81825
N24	111932	P24	129104	T24	132554	H24	264668
N25	105085	P25	147587	T25	105890	H25	133987
N26	149134	P26	126203	T26	115816	H26	73407
N27	77704	P27	82246	T27	81995		
N28	66816	P28	54365	T28	55188		
N29	62763	P29	74825	T29	61295		
N30	91499	P30	91878	T30	83921		
N31	58545	P31	53260	T31	58090		
N32	61960	P32	45183	T32	53948		
N33	50647	P33	55754	T33	62300		
N34	57270	P34	52172	T34	46684		
N35	76050	P35	75587	T35	76693		
N36	75731	P36	57213	T36	98168		
N37	86656	P37	80412	T37	117612		
N38	94949	P38	79039	T38	102098		
N39	79271	P39	89676	T39	87990		

N40	92695
N41	87419
N42	94206
N43	79890
N44	79593
N45	98128
N46	91493
N47	71109
N48	113876
N49	105629
N50	183481
N51	71259
N52	65009
N53	48076
N54	83529
N55	79262
N56	73570
N57	78665
N58	65960
N59	80812
N60	76647
N61	67978
N62	62745
N63	59347
N64	108406
N65	79624
N66	56145
N67	66443
N68	58784
N69	75121
N70	82522
N71	156600
N72	74608
N73	87601
N74	119079
N75	376805

P40	80374
P41	98678
P42	81321
P43	55820
P44	90518
P45	75680
P46	84422
P47	91671
P48	85321
P49	76488
P50	68348
P51	81488
P52	63624
P53	75060
P54	78800
P55	98523
P56	128886
P57	70107
P58	58764
P59	77748
P60	93234
P61	61093
P62	64925
P63	62035
P64	48797
P65	78095
P66	62312
P67	65064
P68	64843
P69	85004
P70	66326
P71	136182
P72	53172
P73	55431
P74	44155
P75	49145

T40	73942
T41	85521
T42	91742
T43	84455
T44	87092
T45	86572
T46	80245
T47	163751
T48	120190
T49	93642
T50	61726
T51	62263
T52	53335
T53	68244
T54	72574
T55	96886
T56	61288
T57	32690
T58	70372
T59	52696
T60	81381
T61	66964
T62	56034
T63	60790
T64	60211
T65	76460
T66	74047
T67	89766
T68	71874
T69	56982
T70	68216
T71	119913
T72	35352
T73	53206
T74	63174
T75	51961
