# Supplementary material for: Differential Mucosal Microbiome Profiles across Stages of Human Colorectal Cancer
Source: Life (Basel). 2021 Aug 13;11(8):831. doi: 10.3390/life11080831 (PMC8401903; doi:10.3390/life11080831)
Supplement: Supplementary file 1 [file life-11-00831-s001.zip › Supplementary Table 4.pdf]

Supplementary Table Summary of bacterial taxa identified in four groups shown in Figure 4.

Group I: stage I > II > III

Bacterium	Metabolism and pathogenicity	Reference
Bacteroides	*Bacteroides* are dominant beneficial bacteria, which metabolize polysaccharides and oligosaccharides, providing nutrition and vitamins to the host and other intestinal microbial residents. Some strains, e.g. *Bacteroides fragilis*, produce *B. fragilis* toxin which causes Inflammatory bowel disease.	1, 2
Prevotella	Intestinal *Prevotella* colonization results in metabolic changes in the microbiota, which reduce IL-18 production and consequently exacerbate intestinal inflammation and potential systemic autoimmunity.	3
Ruminococcus	*Ruminococcus* belongs to probiotics and expresses mucus-, fucose-, and human milk oligosaccharides (HMO)-related degradation enzymes.	4

Dorea	Abundance of *Dorea* increases in dysbiosis of gut microbiota caused by other disease, such as esophageal cancer and nonalcoholic fatty liver disease.	5, 6
Dialister	*Dialister* is Gram-stain-negative in healthy faecal sample, and utilizes succinate and causes repetitive bartholinitis episodes.	7, 8
Faecalibacterium	*Faecalibacterium* plays a major role in the regulation of gut barrier, inflammation and metabolic functions.	9
Coprococcus	*Coprococcus* produces butyrate, and β-Glucan is a major growth substrate for *Coprococcus eutactus*.	10, 11
Sutterella	*Sutterella* is associated with gastrointestinal diseases. *Sutterella* does not induce substantial inflammation, but has a capacity to degrade IgA.	12
Selenomonas	*Selenomonas* is closely associated with CRC patients with hyperlipoidaemia.	13
Eubacterium	Compared to healthy samples, abundance of *Eubacterium* decreases in patients with IBD and CRC.	14, 15

Eggerthella	*Eggerthella lenta* is involved in converting reaction of Plant-derived lignans in food to enterolignans2.	16
Phascolarctobacterium	*Phascolarctobacterium* can produce short-chain fatty acids, including acetate and propionate, and is associated with the metabolic state and mood of the host.	17
Desulfovibrio	*Desulfovibrio* is commensal microbe colonising the mucus gel layer of the colon, metabolises the sulfate moiety of sulfated mucins, and has increased affinity to ulcerative colitis mucin.	18
Comamonas	*Comamonas* abundance changes when resistant starch intervention is used to reduce body fat.	19
Anaerostipes	*Anaerostipes* converts lactate-acetate and sugar to butyrate.	20
Paraprevotella	*Paraprevotella* is positively correlated with damaged gut integrity induced by major depressive disorder.	21
Neisseria	Anti-inflammatory microenvironment causes decreased *Neisseria* abundance.	22
Slackia	*Slackia* is gut-associated bacteria that play roles in host lipid and xenobiotic metabolism. *Slackia* may be free living in the lumen because it has low adhesion to cells.	23

Group II: stage I < II < III

Bacterium	Metabolism and pathogenicity	Reference
Fusobacterium	*Fusobacterium* has high abundance in intestine in CRC patients, and low abundance in health intestine.	24
Delftia	*Delftia* contains IMP-1 Metallo-β-Lactamase which is resistant to antibacterial activity against β-Lactams. *Delftia* is associated with Endophthalmitis and pneumonia with lung cavities formation.	25, 26, 27
Citrobacter	*Citrobacter* is able to induce colitis.	28
Shewanella	*Shewanella* is found in water environment and in patients, and can act as theoriginator of oxacillinase in Gram-negative bacteria.	29
Klebsiella	*Klebsiella* induces Pneumosepsis, produces cytotoxin, and is associated with necrotizing enterocolitis.	30, 31

Granulicatella	*Granulicatella* causes infective endocarditis and endophthalmitis.	32, 33
Capnocytophaga	*Capnocytophaga* causes infection in wound.	34
Pseudomonas	*Pseudomonas* produces exopolysaccharides and infection.	35, 36
Pyramidobacter	*Pyramidobacter* produces acetic and isovaleric acids.	37
Acinetobacter	*Acinetobacter* relates to carnitine metabolism.	38
TG5	Unknown	
Moryella	*Moryella* is weakly saccharolytic and produces indole, acetate, butyrate and lactate as major metabolic end products.	39
Stenotrophomonas	*Stenotrophomonas* is opportunistic emergent pathogen causing hospital-acquired infections.	40
Megasphaera	A butyrate-producing bacterium	41
Eikenella	*Eikenella* produces lysine decarboxylase.	42
Vibrio	*Vibrio* uses Lysine succinylation as post-translational modification to modulate host factors critical for infection.	43

Turicibacter	*Turicibacter* is an anaerobic, Gram-positive bacterium, isolated from human feces.	44
Mycobacterium	*Mycobacterium* infection is found in patients with metastatic colorectal cancer.	45
Dickeya	*Dickeya* relates to potato infection.	46
Rubellimicrobium	*Rubellimicrobium* exists in the soil.	47
Plesiomonas	*Plesiomonas* has several virulence factors, such as lysophospholipase, a twin-arginine translocation system and the type VI secretion effector Phospholipase A1, which relate to diarrhoeal disease.	48
Rubrobacter	*Rubrobacter* produces D-Amino Acid Oxidase.	49
Erwinia	*Erwinia* has high abundance in intestine of gastroenteritis patients.	50
Dysgonomonas	*Dysgonomonas* is Gram-stain negative cocci. It grows optimally at 37°C and is catalase positive but oxidase negative.	51
Paludibacter	A propionate-producing fermentative bacterium	52

Group III: stage I > II < III

Bacterium	Metabolism and pathogenicity	Reference
Akkermansia	Bacterial biomarker of colorectal cancer	53
Sneathia	*Sneathia amnii* produces a cytotoxin, named CptA for cytopathogenic toxin, component A, which is capable of permeabilizing chorionic trophoblasts and lysing human red blood cells.	54
Morganella	*Morganella* produces extended-spectrum β-lactamase, and causes wound infections after colorectal surgery.	55
Parabacteroides	*Parabacteroides* has higher abundance in tumor tissues than adjacent normal tissues in CRC patients.	56
Ruminococcus	Dietary α-ketoglutarate intervention may protect against inflammation-related CRC and increases *Ruminococcus* population.	57
Porphyromonas	*Porphyromonas gingivalis* is a keystone pathogen in periodontitis and promotes the proliferation of colorectal cancer cells by activating the MAPK/ERK signaling pathway.	58

Bilophila	*Bilophila* accesses sulfite from taurine to produce hydrogen sulfide.	59
Gemella	*Gemella* is associated with CRC. The risk of CRC is increased in patients with bacteremia from *Gemella*.	60
Haemophilus	*Haemophilus* was depleted in the transition from stage 0 to early-stage CRC.	61
Leptotrichia	*Leptotrichia* is significant co-occurrence within individual tumors.	62
Odoribacter	The relative abundance of *Odoribacter* is significantly increased in CRC gut flora after 5-Fluorouracil treatment	63
Acidaminococcus	Increased relative abundance of *Acidaminococcus* is associated with future linear growth deficits.	64
Butyricimonas	A closely interaction between *Butyricimonas* and *Clostridium* is observed in the microbiome network in CRC samples.	65
Anaerotruncus	The relative abundances of *Anaerotruncus* displayed significant relationships with both steroid biosynthesis and terpenoid and triterpenoid biosynthesis pathways.	66

Christensenella	The abundance of longevity related *Christensenella* species in gut microbiota increases after fasting and is inversely correlated with age as well as body mass index.	67
WAL_1855D	Unknown	

Group IV: stage I < II >III

Bacterium	Metabolism and pathogenicity	Reference
Streptococcus	*Streptococcus bovis* contributes to the development of CRC via recruiting CD11b^{+}TLR-4^{+} cells, and should be investigated for early detection of colorectal pathology. *Streptococcus gallolyticus subsp. gallolyticus* (*Sgg*) infection has gained considerable attention for its strong association with CRC.	68, 69, 70
Parvimonas	Bacterial biomarker of colorectal cancer	53
Peptostreptococcus	Bacterial biomarker of colorectal cancer	53
Oscillospira	High *Oscillospira* abundance indicates constipation and low BMI.	71

Blautia	*Blautia* is inversely associated with visceral fat area.	72
Bifidobacterium	A possible role of *Bifidobacteria* in determining distinct tumor characteristics or as an indicator of dysfunctional mucosal barrier in CRC. *Bifidobacterium longum* suppresses colorectal carcinogenesis.	73, 74
Campylobacter	*Campylobacter jejuni* produces a genotoxin, cytolethal distending toxin, which has DNAse activity and causes DNA double-strand breaks and promotes colorectal tumorigenesis.	75
Roseburia	*Roseburia* produces butyrate and shows a significant reduction in the gut microbiota of CRC rats.	76
Collinsella	*Collinsella* is proinflammatory bacterium, and is associated with biopsy-proven nonalcoholic steatohepatitis.	77
Bulleidia	*Bulleidia* is an anaerobic Gram-positive pathogen identified within oral and gastroenterological ecological niches.	78
Megamonas	*Megamonas* is associated with lymph node metastasis status.	79
Veillonella	*Veillonella* is more abundant in the proximal segments than in the distal segments of the colon.	80

Prevotella	*Prevotella* produces butyrate and reduces abundance in CRC patients.	81
Enterococcus	Some literatures suggest *Enterococcus* has a harmful role, whereas other papers report *Enterococcus faecalis* as an important probiotic microorganism with great applicability in food products.	82
Clostridium	*Clostridium* causes clostridium difficile infection after colorectal surgeries.	83
Lactobacillus	As a probiotic, *Lactobacillus* prevents dimethylhydrazine-induced colorectal cancers.	84
Serratia	Prodigiosin isolated from cell wall of *Serratia marcescens* alters expression of apoptosis-related genes and increases apoptosis in colorectal cancer cells.	85
Actinomyces	*Actinomyces* is significantly increased in multiple polypoid adenomas and intramucosal carcinomas.	86
Epulopiscium	Unknown	
Bradyrhizobium	*Bradyrhizobium* has higher abundance in off-tumor site of CRC intestine than on-tumor site or healthy person.	15

Anaerococcus	*Anaerococcus* is significantly increased in moderately severe acute pancreatitis.	87
Dethiosulfatibacter	*Dethiosulfatibacter* has higher abundance in on-tumor site of CRC intestine than off-tumor site or healthy person.	15
Peptoniphilus	*Peptoniphilus* is a Gram-positive anaerobic coccus mainly involved in polymicrobial infections, and is reported in a case of peritoneal infection in a patient with intestinal occlusion	88
Lachnospira	*Lachnospira* shows a negative association with asthma risk under 4 years of age.	89
Mogibacterium	*Mogibacterium* causes IL8/IFNγ/TNFα pro-inflammatory effect.	90
Schwartzia	*Schwartzia* shows higher abundance in off-tumor site of CRC intestine than on-tumor site or healthy person.	15
Proteus	*Proteus* induces a 2-fold increase of VIM gene expression within Caco2 cells after cocultivation with *Proteus vulgaris*, suggesting that *Proteus vulgaris* promotes epithelial-mesenchymal transition.	91

Corynebacterium	*Corynebacterium* has relative abundance in the invasive CRC group compared with early CRC group.	92
Aggregatibacter	*Aggregatibacter* is positively correlated with visceral fat, fasting plasma insulin, and HOMA-IR in non-alcoholic fatty liver disease.	93
Catenibacterium	Compared to normal samples, *Catenibacterium* is only detected in CRC tumor samples	94
Ralstonia	Harmful bacteria for intestine	95
Methylobacterium	*Methylobacterium* shows higher abundance in off-tumor site of CRC intestine than on-tumor site or healthy person.	15
Sphingomonas	*Sphingomonas* shows high abundance in pro-inflammatory intestinal microenvironment.	96

Reference

1. Zafar H,Saier MH, Gut Bacteroides species in health and disease, Gut Microbes, 2021, 13, 1, 1-20.
2. Rabizadeh S, Rhee KJ, Wu SG, et al., Enterotoxigenic Bacteroides fragilis: A Potential Instigator of Colitis, Inflamm Bowel Dis. 2007, 13(12): 1475-83.
3. Iljazovic A, Roy U, Gálvez EJC, et al., Perturbation of the gut microbiome by Prevotella spp. enhances host susceptibility to mucosal inflammation, Mucosal Immunol, 2021, 14, 1, 113-24.
4. Nilsen M, Madelen Saunders C, Leena Angell I, et al., Butyrate Levels in the Transition from an Infant- to an Adult-Like Gut Microbiota Correlate with Bacterial Networks Associated with Eubacterium Rectale and Ruminococcus Gnavus, Genes (Basel), 2020, 11, 11, 1245.
5. Deng Y, Tang D, Hou P, et al., Dysbiosis of gut microbiota in patients with esophageal cancer, Microb Pathog, 2021, 150, 104709.
6. Li F, Ye J, Shao C, et al., Compositional alterations of gut microbiota in nonalcoholic fatty liver disease patients: a systematic review and Meta-analysis, Lipids Health Dis, 2021, 20, 1, 22.
7. Sakamoto M, Ikeyama N, Toyoda A, et al., Dialister hominis sp. nov., isolated from human faeces, Int J Syst Evol Microbiol, 2020, 70, 1, 589-95.
8. Cobo F, Rodríguez-Granger J, Sampedro A, et al., Bartholin's abscess due to Dialister micraerophilus in a woman presenting with repetitive bartholinitis episodes, Med Mal Infect, 2018, 48, 3, 225-6.
9. Moosavi SM, Akhavan Sepahi A, Mousavi SF, et al., The effect of Faecalibacterium prausnitzii and its extracellular vesicles on the permeability of intestinal epithelial cells and expression of PPARs and ANGPTL4 in the Caco-2 cell culture model, J Diabetes Metab Disord, 2020, 19, 2, 1061-9.
10. Altemani F, Barrett HL, Gomez-Arango L, et al., Pregnant women who develop preeclampsia have lower abundance of the butyrate-producer Coprococcus in their gut microbiota, Pregnancy Hypertens, 2021, 23, 211-9.
11. Alessi AM, Gray V, Farquharson FM, et al., β-Glucan is a major growth substrate for human gut bacteria related to Coprococcus eutactus, Environ Microbiol, 2020, 22, 6, 2150-64.
12. Kaakoush NO, Sutterella Species, IgA-degrading Bacteria in Ulcerative Colitis, Trends Microbiol, 2020, 28, 7, 519-22.
13. Han S, Pan Y, Yang X, et al., Intestinal microorganisms involved in colorectal cancer complicated with dyslipidosis, Cancer Biol Ther, 2019, 20, 1, 81-9.
14. Zhang SL, Wang SN, Miao CY, Influence of microbiota on intestinal immune system in ulcerative colitis and its intervention, Front Immunol, 2017, 8, 1674.
15. Wang YJ, Zhang CZ, Hou SB, et al., Analyses of potential driver and passenger bacteria in human colorectal cancer, Cancer Management and Research, 2020, 12, 11553-61.
16. Bess EN, Bisanz JE, Yarza F, et al., Genetic basis for the cooperative bioactivation of plant lignans by Eggerthella lenta and other human gut bacteria, Nat Microbiol, 2020, 5, 1, 56-66.
17. Wu F, Guo X, Zhang J, et al., Phascolarctobacterium faecium abundant colonization in human gastrointestinal tract, Exp Ther Med, 2017, 14, 4, 3122-6.
18. Earley H, Lennon G, Balfe A, et al., A Preliminary Study Examining the Binding Capacity of Akkermansia muciniphila and Desulfovibrio spp., to Colonic Mucin in Health and Ulcerative Colitis, PLoS One, 2015, 10, 10, e0135280.
19. Zhang L, Ouyang Y, Li H, et al., Metabolic phenotypes and the gut microbiota in response to dietary resistant starch type 2 in normal-weight subjects: a randomized crossover trial, Sci Rep, 2019, 9, 1, 4736.
20. Shetty SA, Boeren S, Bui TPN, et al., Unravelling lactate-acetate and sugar conversion into butyrate by intestinal Anaerobutyricum and Anaerostipes species by comparative proteogenomics, Environ Microbiol, 2020, 22, 11, 4863-75.
21. Liśkiewicz P, Kaczmarczyk M, Misiak B, et al., Analysis of gut microbiota and intestinal integrity markers of inpatients with major depressive disorder, Prog Neuropsychopharmacol Biol Psychiatry, 2021, 106, 110076.
22. Rodríguez-García C, Sánchez-Quesada C, Algarra I, et al., The High-Fat Diet Based on Extra-Virgin Olive Oil Causes Dysbiosis Linked to Colorectal Cancer Prevention, Nutrients, 2020, 12, 6, 1705.
23. Cho GS, Ritzmann F, Eckstein M, et al., Quantification of Slackia and Eggerthella spp. in Human Feces and Adhesion of Representatives Strains to Caco-2 Cells, Front Microbiol, 2016, 7, 658.
24. Abed J, Emgård JEM, Zamir G, et al., Fap2 Mediates Fusobacterium nucleatum Colorectal Adenocarcinoma Enrichment by Binding to Tumor-Expressed Gal-GalNAc, Cell Host Microbe, 2016, 20, 2, 215-25.
25. Cho SM, Hong SG, Lee Y, et al., First Identification of IMP-1 Metallo-β-Lactamase in Delftia tsuruhatensis Strain CRS1243 Isolated From a Clinical Specimen, Ann Lab Med, 2021, 41, 4, 436-8.

26. Deb AK, Chavhan P, Chowdhury SS, et al., Endophthalmitis due to Delftia acidovorans: An unusual ocular pathogen, Indian J Ophthalmol, 2020, 68, 11, 2591-4.
27. Yildiz H, Sünnetçioğlu A, Ekin S, et al., Delftia acidovorans pneumonia with lung cavities formation, Colomb Med (Cali), 2019, 50, 3, 215-21.
28. Vong L, Pinnell LJ, Määttänen P, et al., Selective enrichment of commensal gut bacteria protects against Citrobacter rodentium-induced colitis, Am J Physiol Gastrointest Liver Physiol, 2015, 309, 3, G181-92.
29. Leangapichart T, Hadjadj L, Gautret P, et al., Comparative genomics of two Shewanella xiamenensis strains isolated from a pilgrim before and during travels to the Hajj, Gut Pathog, 2021, 13, 1, 9.
30. Wu T, Xu F, Su C, et al., Alterations in the Gut Microbiome and Cecal Metabolome During Klebsiella pneumoniae-Induced Pneumosepsis, Front Immunol, 2020, 11, 1331.
31. Paveglio S, Ledala N, Rezaul K, et al., Cytotoxin-producing Klebsiella oxytoca in the preterm gut and its association with necrotizing enterocolitis, Emerg Microbes Infect, 2020, 9, 1, 1321-9.
32. Alkandari SA, Bhardwaj RG, Ellepola A, et al., Proteomics of extracellular vesicles produced by Granulicatella adiacens, which causes infective endocarditis, PLoS One, 2020, 15, 11, e0227657.
33. Al-Lozi A, Cai S, Chen X, et al., Granulicatella Adiacens as an Unusual Cause of Microbial Keratitis and Endophthalmitis: A Case Series and Literature Review, Ocul Immunol Inflamm, 2021, 1-5.
34. Markewitz RDH, Graf T, Capnocytophaga canimorsus Infection, N Engl J Med, 2020, 383, 12, 1167.
35. Jennings LK, Dreifus JE, Reichhardt C, et al., Pseudomonas aeruginosa aggregates in cystic fibrosis sputum produce exopolysaccharides that likely impede current therapies, Cell Rep, 2021, 34, 8, 108782.
36. Askarian F, Uchiyama S, Masson H, et al., The lytic polysaccharide monooxygenase CbpD promotes Pseudomonas aeruginosa virulence in systemic infection, Nat Commun, 2021, 12, 1, 1230.
37. Downes J, Vartoukian SR, Dewhirst FE, et al., Pyramidobacter piscolens gen. nov., sp. nov., a member of the phylum 'Synergistetes' isolated from the human oral cavity, Int J Syst Evol Microbiol, 2009, 59, Pt 5, 972-80.
38. Massmig M, Reijerse E, Krausze J, et al., Carnitine metabolism in the human gut: characterization of the two-component carnitine monooxygenase CntAB from Acinetobacter baumannii, J Biol Chem, 2020, 295, 37, 13065-78.
39. Carlier JP, K'ouas G, Han XY, Moryella indoligenes gen. nov., sp. nov., an anaerobic bacterium isolated from clinical specimens, Int J Syst Evol Microbiol, 2007, 57, Pt 4, 725-9.
40. Ince N, Yekenkurul D, Danış A, et al., An evaluation of six-year Stenotrophomonas maltophilia infections in a university hospital, Afr Health Sci, 2020, 20, 3, 1118-23.
41. Maki JJ, Looft T, Megasphaera stantonii sp. nov., a butyrate-producing bacterium isolated from the cecum of a healthy chicken, Int J Syst Evol Microbiol, 2018, 68, 11, 3409-15.
42. Levine M, Collins LM, Lohinai Z, Zinc chloride inhibits lysine decarboxylase production from Eikenella corrodens in vitro and its therapeutic implications, J Dent 2021, 104, 103533.
43. Zeng F, Pang H, Chen Y, et al., First Succinylome Profiling of Vibrio alginolyticus Reveals Key Role of Lysine Succinylation in Cellular Metabolism and Virulence, Front Cell Infect Microbiol, 2020, 10, 626574.
44. Cuív PÓ, Klaassens ES, Durkin AS, et al., Draft genome sequence of Turicibacter sanguinis PC909, isolated from human feces, J Bacteriol, 2011, 193, 5, 1288-9.
45. Hirashima T, Nagai T, Shigeoka H, et al., Comparison of the clinical courses and chemotherapy outcomes in metastatic colorectal cancer patients with and without active Mycobacterium tuberculosis or Mycobacterium kansasii infection: a retrospective study, BMC Cancer, 2014, 14, 770.
46. Ge T, Ekbataniamiri F, Johnson SB, et al., Interaction between Dickeya dianthicola and Pectobacterium parmentieri in Potato Infection under Field Conditions, Microorganisms, 2021, 9, 2.
47. Riedel T, Spring S, Fiebig A, et al., Genome sequence of the pink to light reddish-pigmented Rubellimicrobium mesophilum type strain (DSM 19309(T)), a representative of the Roseobacter group isolated from soil, and emended description of the species, Stand Genomic Sci, 2014, 9, 3, 902-13.
48. Edwards MS, McLaughlin RW, Li J, et al., Putative virulence factors of Plesiomonas shigelloides, Antonie Van Leeuwenhoek, 2019, 112, 12, 1815-26.
49. Takahashi S, Furukawara M, Omae K, et al., A Highly Stable D-Amino Acid Oxidase of the Thermophilic Bacterium Rubrobacter xylanophilus, Appl Environ Microbiol 2014, 80, 23, 7219-29.
50. Mori K, Konishi N, Suzuki Y, et al., Comparison between patients with norovirus-related gastroenteritis and asymptomatic carriers with respect to distribution of antibody-complexed viral particles and intestinal flora, J Med Virol, 2018, 90, 12, 1882-7.

51. Bilen M, Fonkou MDM, Dubourg G, et al., Dysgonomonas massiliensis sp. nov., a new species isolated from the human gut and its taxonogenomic description, Antonie Van Leeuwenhoek, 2019, 112, 6, 935-45.
52. Qiu YL, Kuang XZ, Shi XS, et al., Paludibacter jiangxiensis sp. nov., a strictly anaerobic, propionate-producing bacterium isolated from rice paddy field, Arch Microbiol, 2014, 196, 3, 149-55.
53. Osman MA, Neoh HM, Ab Mutalib NS, et al., Parvimonas micra, Peptostreptococcus stomatis, Fusobacterium nucleatum and Akkermansia muciniphila as a four-bacteria biomarker panel of colorectal cancer, Sci Rep, 2021, 11, 1, 2925.
54. Gentile GL, Rupert AS, Carrasco LI, et al., Identification of a Cytopathogenic Toxin from Sneathia amnii, J Bacteriol, 2020, 202, 13, doi:10.1128/JB.00162-20.
55. Kalakouti E, Simillis C, Pellino G, et al., Characteristics of Surgical Site Infection Following Colorectal Surgery in a Tertiary Center: Extended-spectrum β-Lactamase-producing Bacteria Culprits in Disease, Wounds 2017, 30, 4, 108-113.
56. Sheng QS, He KX, Li JJ, et al., Comparison of Gut Microbiome in Human Colorectal Cancer in Paired Tumor and Adjacent Normal Tissues, Onco Targets Ther, 2020, 13, 635-646.
57. Li S, Fu C, Zhao Y, et al., Intervention with α-Ketoglutarate Ameliorates Colitis-Related Colorectal Carcinoma via Modulation of the Gut Microbiome, Biomed Res Int, 2019, 2019, 8020785.
58. Mu W, Jia Y, Chen X, et al., Intracellular Porphyromonas gingivalis Promotes the Proliferation of Colorectal Cancer Cells via the MAPK/ERK Signaling Pathway, Front Cell Infect Microbiol, 2020, 10, 584798.
59. Peck SC, Denger K, Burrichter A, et al., A glycyl radical enzyme enables hydrogen sulfide production by the human intestinal bacterium Bilophila wadsworthia, Proc Natl Acad Sci U S A, 2019, 116, 8, 3171-6.
60. Kwong TNY, Wang X, Nakatsu G, et al., Association Between Bacteremia From Specific Microbes and Subsequent Diagnosis of Colorectal Cancer, Gastroenterology, 2018, 155, 2, 383-390.e8.
61. Yang TW, Lee WH, Tu SJ, et al., Enterotype-based Analysis of Gut Microbiota along the Conventional Adenoma-Carcinoma Colorectal Cancer Pathway, Sci Rep, 2019, 9, 1, 10923.
62. Warren RL, Freeman DJ, Pleasance S, et al., Co-occurrence of anaerobic bacteria in colorectal carcinomas, Microbiome, 2013, 1, 1, 16.
63. Yuan L, Zhang S, Li H, et al., The influence of gut microbiota dysbiosis to the efficacy of 5-Fluorouracil treatment on colorectal cancer, Biomed Pharmacother, 2018, 108, 184-93.
64. Gough EK, Stephens DA, Moodie EE, et al., Linear growth faltering in infants is associated with Acidaminococcus sp. and community-level changes in the gut microbiota, Microbiome, 2015, 3, 24.
65. Zhang Q, Zhao H, Wu D, et al., A comprehensive analysis of the microbiota composition and gene expression in colorectal cancer, BMC Microbiol, 2020, 20, 1, 308.
66. Loke MF, Chua EG, Gan HM, et al., Metabolomics and 16S rRNA sequencing of human colorectal cancers and adjacent mucosa, PLoS One, 2018, 13, 12, e0208584.
67. Lilja S, Stoll C, Krammer U, et al., Five Days Periodic Fasting Elevates Levels of Longevity Related Christensenella and Sirtuin Expression in Humans, Int J Mol Sci, 2021, 22, 5, 2331.
68. Deng Q, Wang C, Yu K, et al., Streptococcus bovis Contributes to the Development of Colorectal Cancer via Recruiting $CD11b^{+}TLR\text{-}4^{+}$ Cells, Med Sci Monit, 2020, 26, e921886.
69. Ng SC, Wong HK, So CK, et al., Streptococcus bovis bacteraemia should be investigated for early detection of colorectal pathology, Hong Kong Med J, 2019, 25, 5, 414.
70. Lin Q, Ye X, Yang B, et al., Real-time fluorescence loop-mediated isothermal amplification assay for rapid and sensitive detection of Streptococcus gallolyticus subsp. gallolyticus associated with colorectal cancer, Anal Bioanal Chem, 2019, 411, 26, 6877-87.
71. Chen YR, Zheng HM, Zhang GX, et al., High Oscillospira abundance indicates constipation and low BMI in the Guangdong Gut Microbiome Project, Sci Rep, 2020, 10, 1, 9364.
72. Ozato N, Saito S, Yamaguchi T, et al., Blautia genus associated with visceral fat accumulation in adults 20-76 years of age, NPJ Biofilms Microbiomes, 2019, 5, 1, 28.
73. Kosumi K,Hamada T,Koh H, et al., The Amount of Bifidobacterium Genus in Colorectal Carcinoma Tissue in Relation to Tumor Characteristics and Clinical Outcome, Am J Pathol, 2018, 188, 12, 2839-52.
74. Fahmy CA,Gamal-Eldeen AM,El-Hussieny EA, et al., Bifidobacterium longum Suppresses Murine Colorectal Cancer through the Modulation of oncomiRs and Tumor Suppressor miRNAs, Nutr Cancer, 2019, 71, 4, 688-700.
75. He Z, Gharaibeh RZ, Newsome RC, et al., Campylobacter jejuni promotes colorectal tumorigenesis through the action of cytolethal distending toxin, Gut, 2019, 68, 2, 289-300.
76. Zhu Q, Jin Z, Wu W, et al., Analysis of the intestinal lumen microbiota in an animal model of colorectal cancer, PLoS One, 2014, 9, 6, e90849.

77. Astbury S, Atallah E, Vijay A, et al., Lower gut microbiome diversity and higher abundance of proinflammatory genus Collinsella are associated with biopsy-proven nonalcoholic steatohepatitis, Gut Microbes, 2020, 11, 3, 569-580.
78. Morgan MA, Goldstein EJ, Bulleidia extructa: An underappreciated anaerobic pathogen, Anaerobe, 2021, 69, 102339.
79. Han S, Wu W, Da M, et al., Adequate Lymph Node Assessments and Investigation of Gut Microorganisms and Microbial Metabolites in Colorectal Cancer, Onco Targets Ther, 2020, 13, 1893-1906.
80. Sheng Q, Du H, Cheng X, et al., Characteristics of fecal gut microbiota in patients with colorectal cancer at different stages and different sites, Oncol Lett, 2019, 18, 5, 4834-44.
81. Huang CC, Shen MH, Chen SK, et al., Gut butyrate-producing organisms correlate to Placenta Specific 8 protein: Importance to colorectal cancer progression, J Adv Res, 2020, 22, 7-20.
82. 82. de Almeida CV,Taddei A,Amedei A, et al., The controversial role of Enterococcus faecalis in colorectal cancer, Therap Adv Gastroenterol, 2018, 11, 1756284818783606.
83. Calu V, Toma EA, Enciu O, et al., Clostridium difficile Infection and Colorectal Surgery: Is There Any Risk?, Medicina (Kaunas), 2019, 55, 10, 683.
84. Dong Y,Zhu J,Zhang M, et al., Probiotic Lactobacillus salivarius Ren prevent dimethylhydrazine-induced colorectal cancer through protein kinase B inhibition, Appl Microbiol Biotechnol, 2020, 104, 17, 7377-89.
85. Hassankhani R, Sam MR, Esmaeilou M, et al., Prodigiosin isolated from cell wall of Serratia marcescens alters expression of apoptosis-related genes and increases apoptosis in colorectal cancer cells, Med Oncol, 2015, 32, 1, 366.
86. Yachida S, Mizutani S, Shiroma H, et al., Metagenomic and metabolomic analyses reveal distinct stage-specific phenotypes of the gut microbiota in colorectal cancer, Nat Med, 2019, 25, 6, 968-76.
87. Yu S, Xiong Y, Xu J, et al., Identification of Dysfunctional Gut Microbiota Through Rectal Swab in Patients with Different Severity of Acute Pancreatitis, Dig Dis Sci, 2020, 65, 11, 3223-37.
88. Cobo F, Rodríguez-Granger J, Sampedro A, et al., Peritoneal infection due to Peptoniphilus harei in a patient with intestinal occlusion, Anaerobe, 2017, 44, 126-7.
89. Stiemsma LT, Arrieta MC, Dimitriu PA, et al., Shifts in Lachnospira and Clostridium sp. in the 3-month stool microbiome are associated with preschool age asthma, Clin Sci (Lond), 2016, 130, 23, 2199-2207.
90. Li Q, Pu Y, Lu H, et al., Porphyromonas, Treponema, and Mogibacterium promote IL8/IFNγ/TNFα-based pro-inflammation in patients with medication-related osteonecrosis of the jaw, J Oral Microbiol, 2020, 13, 1, 1851112.
91. Wachsmannova L, Stevurkova V, Ciernikova S, Changes in SNAI1 and VIM gene expression in Caco2 cells after cocultivation with bacteria from colorectal cancer biopsies, Neoplasma, 2019, 66, 2, 271-5.
92. Zorron Cheng Tao Pu L, Yamamoto K, Honda T, et al., Microbiota profile is different for early and invasive colorectal cancer and is consistent throughout the colon, J Gastroenterol Hepatol, 2020, 35, 3, 433-7.
93. Komazaki R, Katagiri S, Takahashi H, et al., Periodontal pathogenic bacteria, Aggregatibacter actinomycetemcomitans affect non-alcoholic fatty liver disease by altering gut microbiota and glucose metabolism, Sci Rep, 2017, 7, 1, 13950.
94. Zhang Q, Zhao H, Wu D, et al., A comprehensive analysis of the microbiota composition and gene expression in colorectal cancer, BMC Microbiol, 2020, 20, 1, 308.
95. Hou Q, Zhao F, Liu W, et al., Probiotic-directed modulation of gut microbiota is basal microbiome dependent, Gut Microbes, 2020, 12, 1, 1736974.
96. Rodríguez-García C, Sánchez-Quesada C, Algarra I, et al., The High-Fat Diet Based on Extra-Virgin Olive Oil Causes Dysbiosis Linked to Colorectal Cancer Prevention, Nutrients, 2020, 12, 6, 1705.
